# H2A.Z controls the stability and mobility of nucleosomes to regulate expression of the LH genes

**DOI:** 10.1038/ncomms12958

**Published:** 2016-09-22

**Authors:** Sergei Rudnizky, Adaiah Bavly, Omri Malik, Lilach Pnueli, Philippa Melamed, Ariel Kaplan

**Affiliations:** 1 Faculty of Biology, Technion—Israel Institute of Technology, Haifa 32000, Israel; 2 Russell Berrie Nanotechnology Institute, Technion—Israel Institute of Technology, Haifa 32000, Israel

## Abstract

The structure and dynamics of promoter chromatin have a profound effect on the expression levels of genes. Yet, the contribution of DNA sequence, histone post-translational modifications, histone variant usage and other factors in shaping the architecture of chromatin, and the mechanisms by which this architecture modulates expression of specific genes are not yet completely understood. Here we use optical tweezers to study the roles that DNA sequence and the histone variant H2A.Z have in shaping the chromatin landscape at the promoters of two model genes, *Cga* and *Lhb*. Guided by MNase mapping of the promoters of these genes, we reconstitute nucleosomes that mimic those located near the transcriptional start site and immediately downstream (+1), and measure the forces required to disrupt these nucleosomes, and their mobility along the DNA sequence. Our results indicate that these genes are basally regulated by two distinct strategies, making use of H2A.Z to modulate separate phases of transcription, and highlight how DNA sequence, alternative histone variants and remodelling machinery act synergistically to modulate gene expression.

## Abstract (web summary)

Chromatin architecture at promoters can influence gene transcription. Here, the authors use a biophysical approach to elucidate how DNA sequence and histone variant usage modulate the properties of nucleosomes at the promoters of two target genes.

## Graphical abstract

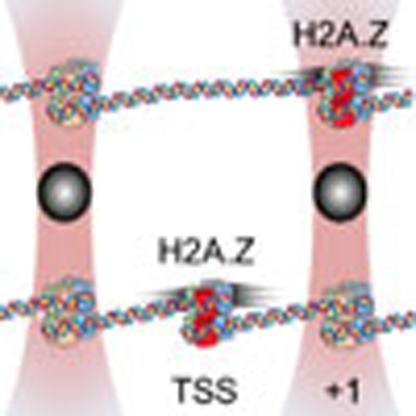

Nucleosomes reduce the accessibility of the transcription machinery to DNA, acting as obstacles for the binding of transcription factors (TFs) and for the subsequent elongation of RNA polymerase II (Pol2). Hence, nucleosomal stability, position and dynamics act as a first and important layer in the complex modulation of gene expression levels. A general picture of how chromatin packaging is tailored at gene promoters to help regulate expression has emerged from genome-wide mapping of nucleosome positions^1^. These studies have revealed that actively transcribed genes often contain nucleosome-depleted regions (NDRs) in the vicinity of their regulatory regions together with a well-positioned +1 nucleosome, and that their nucleosomes have high turnover rates and are enriched with certain histone variants.

Of particular importance is the evolutionarily conserved and essential variant of histone H2A, H2A.Z, which has critical roles in development, differentiation, T-cell activation and more^2^. Interestingly, H2A.Z is enriched at the promoters of both active and silent Pol2-regulated genes^3^. Moreover, contrasting results were reported as to the role of H2A.Z in stabilizing or destabilizing the nucleosome, and to its repressive or activating role in transcription^4^. Although there is a wide consensus on the importance of H2A.Z, the mechanisms by which it influences gene expression have remained elusive.

Clearly, the architecture of promoters is the result of the combined effect of a number of factors, including the sequence of DNA^5^,^6^, post-translational modifications of the histone proteins^7^, competition with TFs^8^, the activity of chromatin remodellers^9^,^10^, histone variant usage^11^,^12^,^13^,^14^,^15^ and more. Yet, the differential contribution of each of the above factors in shaping the chromatin architecture, and the mechanisms by which this architecture modulates the expression levels of specific genes, are not yet completely understood. This is the result, in part, of the complex and dynamic character of chromatin, which presents a challenge for studies with classical ‘bulk' methods, with their inherent ensemble averaging. Previous studies have shown that single-molecule manipulation techniques, such as optical tweezers, can be used to overcome this synchronization problem^16^,^17^,^18^,^19^,^20^,^21^,^22^,^23^. In particular, the Wang lab showed that by force-unwinding the DNA it is possible to generate a detailed histone–DNA interaction energy landscape^24^, to observe the effect of remodellers on the position of nucleosomes^25^ and to study the interplay between remodelling and TF binding^8^.

The *Cga* and *Lhb* genes encode, respectively, the α and β subunits of luteinizing hormone (LH), a glycoprotein secreted by the anterior pituitary gonadotropes, which plays a pivotal role in regulating development and function of the gonads. LH is positively regulated by the hypothalamic gonadotropin-releasing hormone that activates transcription of both genes, involving a number of modifications to the chromatin^26^,^27^,^28^,^29^. Although *Cga* and *Lhb* are both expressed in the gonadotropes under similar hormonal control, the α subunit comprises also a part of the follicle-stimulating hormone, as well as part of the thyroid-stimulating hormone expressed in the thyrotropes; thus, their basal levels of expression differ, with the *Cga* gene being more widely expressed and at higher levels.

In our work we have used single-molecule chromatin ‘unzipping' with high-resolution optical tweezers, in combination with micrococcal nuclease (MNase) mapping of nucleosomal occupancy in cultured cells, to study the roles that DNA sequence and histone variant H2A.Z play in shaping the chromatin landscape at the promoters of *Cga* and *Lhb*. Our aim was to elucidate how the structure and dynamics of chromatin at the promoters of these genes are shaped to achieve their relative expression levels. Our strategy was to use the results of bulk measurements on gonadotropes to inform single-molecule *in vitro* experiments. Hence, guided by MNase mapping of nucleosome positioning at the promoters of *Cga* and *Lhb*, we reconstituted nucleosomes that mimic those located near the transcription start site (TSS) and immediately downstream of the TSS (+1). Then, we measured the forces required to disrupt these nucleosomes, and their position along the DNA sequence. Our results shed light on two alternative strategies for gene regulation.

## Results

### Mapping of nucleosome positioning on *Cga* and *Lhb* genes

**Figure 1:**
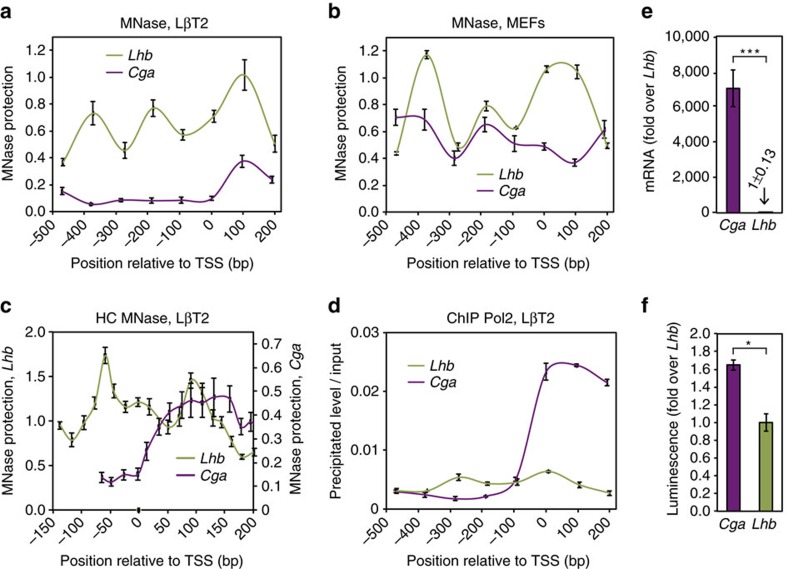
Distinct chromatin structures at the promoters of *Cga* and *Lhb* appear responsible for their expression patterns. (**a**–**c**) Native chromatin in (**a**) LβT2 cells that express both *Cga* and *Lhb*, and (**b**) MEFs that do not express them was digested with MNase and subjected to qPCR (MNase-qPCR) with primers amplifying ∼100 bp non-overlapping fragments spanning ∼−550/+250 bp of each gene. (**c**) For high-coverage nucleosome maps in LβT2 cells, MNase-digested LβT2 DNA was subjected to qPCR with primers amplifying ∼70 bp overlapping fragments. MNase protection (that is, amount of DNA amplified in qPCR reaction after MNase digestion of genomic DNA for each primer pair) is plotted for each amplicon centre relative to genomic coordinates of each gene respective to its TSS. (**d**) ChIP for Pol2 was carried out in LβT2 cells, using the same sets of primers as in **a** and **b**. The levels of precipitated DNA are presented relative to the levels in input samples. (**e**) Total RNA from LβT2 cells was reverse transcribed with random primers before qPCR. RNA levels were quantitated using a standard curve of plasmid *Cga* and *Lhb* cDNA, normalized to RPL0P mRNA and are presented relative to the levels of *Lhb*. Data shown as mean±s.e.m., *n*=3–5; *P*=1.3 × 10^−4^, two-sample Student's *t*-test. ***P<0.001. (**f**) LβT2 cells were transfected with *Cga* (−507/+46) or *Lhb* (−755/+6) DNA sequences fused to the luciferase reporter gene. The levels of firefly luciferase were normalized to those of Renilla luciferase and are expressed as fold over *Lhb*-driven luciferase activity. Data shown as mean±s.e.m., *n*=4; *P*=0.03, two-sample Student's *t*-test. *P<0.05.

To map the occupancy of nucleosomes on *Cga* and *Lhb*, we performed quantitative PCR (qPCR) analysis of MNase-digested DNA (MNase-qPCR). We measured occupancy in mature gonadotrope cells (LβT2) that express both genes (Fig. 1a), and in mouse embryonic fibroblasts (MEFs) (Fig. 1b), where the genes are not expressed. Nucleosome occupancy on the *Lhb* gene in gonadotrope cells shows a well-defined first nucleosome downstream of the TSS region. A similar picture is observed in MEFs, and in MNase-seq experiments performed by others^30^. This occupancy pattern resembles also the one obtained by a sequence-based prediction^31^ (Supplementary Fig. 1a), suggesting that the measured occupancy patterns are dictated mainly by the sequence of DNA. In striking contrast, although in MEFs the mapping of nucleosomes on the *Cga* gene shows significant occupancy and similarity to the prediction, in gonadotrope cells a markedly different occupancy is observed: the *Cga* gene promoter harbours a NDR, and a well-defined +1 nucleosome positioned downstream of the TSS.

To obtain a more precise mapping of the nucleosomes found on *Cga* and *Lhb*, we designed overlapping primers amplifying ∼70 bp fragments spanning regions corresponding to the positions of the nucleosomes found proximal to the TSS. These high-coverage nucleosome maps (Fig. 1c) revealed the positions of TSS and +1 nucleosomes with higher precision: for *Lhb*, a TSS nucleosome was found between −128 and +23. This nucleosome was not revealed by the low-coverage map, probably as a result of the primers used for low-resolution mapping (−42/+58 and −139/−40), which skip over the most probable position as indicated by the high-coverage map. In addition, the +1 nucleosome was positioned immediately downstream of the TSS, between +29 and +179. These positions are consistent, and in good agreement with the DNA-based prediction and the positions in MEF cells. The high-coverage analysis for the *Cga* gene in gonadotropes revealed that the +1 nucleosome is positioned between +39 and +189, with a NDR immediately upstream of it, a result consistent with the previously observed pattern of +1 nucleosome positioning for active promoters^32^.

Interestingly, the high-coverage analysis revealed that the *Cga* +1 nucleosome is positioned further downstream than the +1 nucleosome on *Lhb*. Such a difference could be a consequence of high Pol2 occupancy at the promoter, as proposed in genome-wide studies^32^. Indeed, chromatin immunoprecipitation (ChIP-qPCR) revealed ∼8-fold higher signal of Pol2 levels at the TSS of *Cga* as compared with *Lhb* (Fig. 1d).

Basal *Cga* mRNA levels are ∼7,000-fold higher than those of *Lhb* in cultured mature gonadotrope cells (Fig. 1e), which could be due to numerous effects beyond merely nucleosome positioning. We therefore wanted to check whether the distinct levels of basal expression result from the observed differences in chromatin structure, or are due to other factors in the cells, such as TF availability or activity. To that end, we fused each gene promoter (−507/+46 fragment of *Cga* gene or −755/+6 fragment of *Lhb* gene) to a luciferase reporter and transfected LβT2 cells. The luciferase assay allowed us to look at the activity of these promoters out of their native genomic context and devoid of their native packaging into chromatin. The measured luminescence shows only an ∼1.5-fold higher activity level for the *Cga* promoter over that of the *Lhb* (Fig. 1f). This minor increase cannot explain the 7,000-fold difference in RNA levels between the endogenous genes in these cells, and suggests that the distinct chromatin organization at these two genes is responsible for the major differences observed in their expression.

### Characterization of nucleosomes assembled on *Cga* and *Lhb* DNA

**Figure 2:**
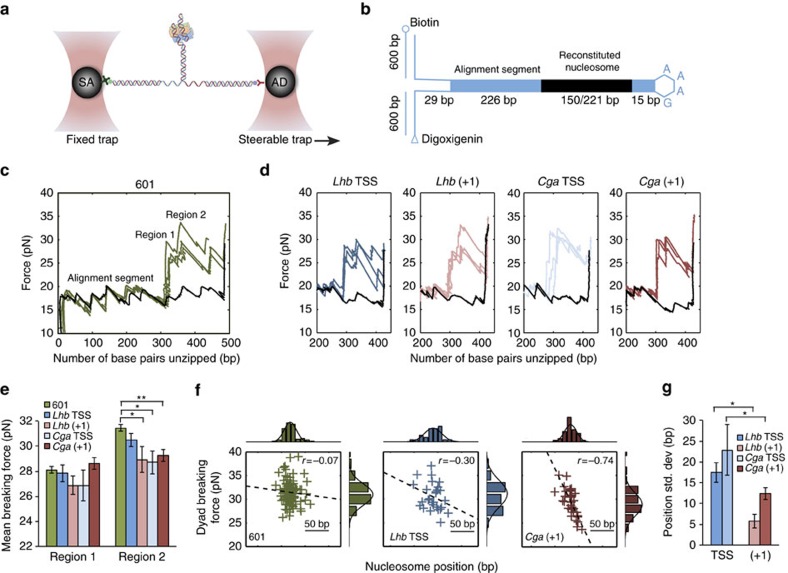
Single-molecule probing of nucleosomes reconstituted on *Cga* and *Lhb* DNA sequences. (**a**) Nucleosomes reconstituted on *Lhb* and *Cga* DNA sequences (150 bp), and a DNA sequence harbouring the 601 DNA-positioning element (221 bp) are connected to dsDNA molecular handles, which are attached to polystyrene beads trapped in two separate optical traps. One of the traps is moved to stretch the tethered construct. (**b**) The reconstituted nucleosome is ligated to a fixed alignment sequence and a short stem-loop that prevents breaking of the tether after unzipping. (**c**) Unzipping curves for nucleosomes reconstituted using the 601 sequence (green) and the ‘naked' (that is, no nucleosome) 601 sequence (black). (**d**) Unzipping curves of nucleosomes reconstituted on the TSS and +1 sequences of *Lhb* and *Cga*, and their respective naked DNA curve. (**e**) Breaking force for region 1 and region 2 interactions, for all probed nucleosomes. Data shown as mean±s.e.m., *n*=88, 28, 8, 7 and 37; *P*=0.002, 0.03 and 0.02, two-sample Kolmogorov–Smirnov test. *P<0.05, **P<0.01. (**f**) Two-dimensional scatter plots of region 2 breaking force versus position, with univariate histograms of the force (top) and position (right). Data shown include all the individual measured unzipping traces for 601, *Lhb* TSS and *Cga* +1 nucleosomes. Gaussian fits to the histograms (solid black) and linear fits to the scatter plot data (dashed black) are drawn to guide the eye. (**g**) Positional dispersion of the *Lhb* and *Cga* nucleosomes. Data shown as region 2 position s.d.±s.e., *n*=28, 8, 7 and 37; *P*=0.02 and 0.02, Ansari–Bradley test. *P<0.05.

The large differences observed in MNase protection for nucleosomes located at the same positions relative to the TSS for the two genes suggest that DNA sequence might affect not only nucleosome position but also stability. Since we also observed well-positioned nucleosomes for *Lhb*, both in MEFs and in gonadotropes, but poor positioning or absence of nucleosomes for *Cga*, we asked whether this might be the result of differences in the strength of nucleosomes formed at these sequences. More generally, TF binding and Pol2 elongation are affected not only by the mere presence of nucleosomes but also by their specific properties, such as the strength of histone–DNA interaction and the positional dynamics of nucleosomes, which we expect to be dictated by the underlying DNA sequence^33^. To gain information on the mapped nucleosomes, beyond the ensemble-averaged position measured by the MNase assays, we characterized each of the relevant nucleosomes at the single-molecule level. We reconstituted promoter proximal nucleosomes on *Cga* and *Lhb* DNA sequences using the results from the high-coverage MNase experiments for *Lhb* and the sequence-based prediction and high-coverage MNase in MEF cells for *Cga* (Supplementary Fig. 1b). We assembled nucleosomes using ∼150 bp for each TSS and +1 nucleosome with mouse canonical histones, which were expressed in *Escherichia coli* to obtain a homogenous population of histone proteins lacking post-translational modifications and histone variants (Supplementary Figs 2a,b and 3a,b). We attached each of the DNA strands on the reconstituted nucleosomes to an ∼600 bp dsDNA ‘handle' harbouring, at its other end, a specific tag that allowed binding of each handle to one of two ∼1 μm polystyrene beads (Fig. 2a,b and Supplementary Fig. 3c,d). Using a double-trap optical tweezers set-up (Fig. 2a), we trapped the two beads with the tethered nucleosome between them and subjected the tether to mechanical force by moving one bead relative to the other with a piezoelectric-controlled mirror mount. As the applied force reached ∼17 pN, consecutive events of sudden force decrease concomitant with extension increase indicate the gradual conversion of dsDNA to ssDNA, and the propagation of the unzipping fork (Fig. 2c). In the presence of a nucleosome, interactions between DNA and histones need also to be disrupted to unzip the DNA, hence a large increase in the force is observed as the unzipping fork reaches the nucleosome. As the force reaches values of 23–35 pN, nucleosomes break down in a clear pattern, which is easily distinguishable from that of naked DNA.

It has been shown^24^,^25^,^34^ that nucleosomes reconstituted on the ‘601' positioning sequence^33^ disassemble under force with a specific signature that reveals the strength of the underlying interactions. Notably, a rip is observed at the nucleosome's dyad that corresponds to interactions with the H3/H4 tetramer, and additional regions of interaction are observed ∼±40 bp with respect to the dyad, which correspond to interactions of the DNA with H2A/H2B dimers. Since destabilizing the H3/H4 interactions leads to nucleosome disassembly, in most unzipping experiments only two interaction regions are observed. The unzipping measurements of nucleosomes reconstituted on the 601 sequence (Fig. 2c and Supplementary Fig. 4) are consistent with these previous studies, although the breaking forces measured are overall smaller by a few pN, as expected given the slightly different buffer conditions and force loading rate.

Nucleosomes reconstituted on the *Cga* and *Lhb* DNA sequences exhibited similar disassembly patterns, with two prominent regions of strong interaction (Fig. 2d and Supplementary Fig. 4). The unzipping curves allow us to determine the breaking forces and the location of the two interaction regions on each probed nucleosome. Our results indicate that although there is a significant difference in mean breaking force between the synthetic high-affinity 601 DNA and the naturally occurring sequences, no significant differences among the latter were observed (Fig. 2e,f). In contrast, dispersion in the position of the nucleosomes, which serves as a measure of the degree of their positioning, revealed a significant effect of the DNA sequence. Both TSS nucleosomes have significantly higher positional dispersion (that is, less defined positioning) as compared with the +1 nucleosomes (*P*=0.025 for *Lhb*, *P*=0.024 for *Cga*; two-sample Ansari–Bradley test; Fig. 2g). As will be discussed below, this dispersion can have a major effect on the gene expression levels.

Interestingly, although an effect for the sequence on the breaking force is not measured when averaged over the ensemble of measured nucleosomes, by looking at individual nucleosomes (Fig. 2f) it is clear that in some cases there is a significant effect for the sequence even at the base-pair scale. For example, at the *Cga* +1 nucleosome, a significant correlation (*r*=0.74, *P*=2.7 × 10^−7^) exists between the precise position of individual nucleosomes and the measured H3/H4 breaking force, which was not observed for the 601 sequence or the *Lhb* TSS. One could speculate that such a correlation may play a regulatory role, for example, allowing remodellers to affect the nucleosome's stability by modifying its position.

Overall, the fact that all four measured nucleosomes are relatively stable, and share comparable breaking forces, raises two questions about the regulation of these genes: first, how is the *Cga* TSS nucleosome evicted in gonadotropes? Second, what mechanism is used to modulate the expression of *Lhb*, without affecting the overall positioning of this nucleosome?

### Establishment of the NDR at the *Cga* promoter

**Figure 3:**
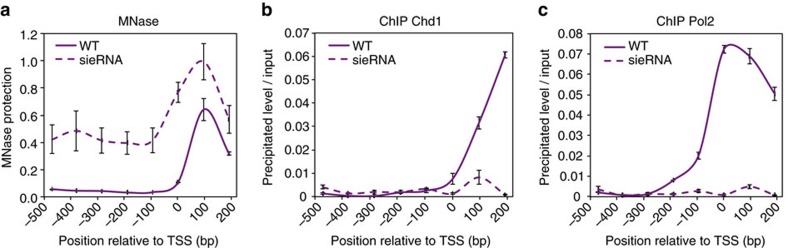
Chd1 may play a role in establishing the *Cga* NDR. (**a**) MNase-qPCR and ChIP for (**b**) Chd1 and (**c**) Pol2 was carried out in αT3-1 wild-type (WT) cells that express *Cga* and in αT3-1 cells stably expressing shRNA against eRNA. MNase-qPCR data are analysed and presented as in Fig. 1b, and for ChIP data are as in Fig. 1d. Data are shown as mean±s.e.m., *n*=2–3.

The fact that a nucleosome can form at the *Cga* TSS, and that this nucleosome exhibits a similar disassembly pattern as the other nucleosomes measured suggests, as previously shown^6^, that the NDR found *in vivo* is not the result of a DNA sequence that prevents the formation of a nucleosome, but is formed by the action of remodelling machinery. One such remodeller is the chromodomain-helicase-DNA-binding protein 1 (Chd1), whose activity is required to maintain nucleosome occupancy within promoter regions, as seen in genome-wide studies in MEFs^35^. Chd1 was found associated with the *Cga* promoter in gonadotropes, dependent on the presence of a distal enhancer non-coding RNA (eRNA) that is essential for active chromatin at this promoter^29^. To check whether the NDR in *Cga* is dependent on Chd1 recruitment, we performed MNase-qPCR in gonadotrope cells stably transfected with shRNA against the eRNA. This manipulation allowed a gene-specific perturbation of the recruitment of Chd1 to *Cga*, instead of knocking down Chd1 directly, which, due to the widespread role^35^ of Chd1, could lead to indirect effects. The nucleosome occupancy pattern in eRNA knockdown cells (Fig. 3a) showed an ∼7-fold increase in MNase protection in the region immediately upstream of the TSS and a less positioned +1 nucleosome, as compared with wild-type cells, indicating that eRNA is involved in the creation of the NDR. ChIP with an antibody against Chd1 showed ∼6-fold decrease in Chd1 occupancy at the promoter of *Cga* (Fig. 3b), to a level similar to the one measured for *Lhb* (Supplementary Fig. 6), consistent with previously published results^29^. In addition, ChIP with an antibody against Pol2 showed ∼70-fold decrease in the total Pol2 binding to the TSS (Fig. 3c). Taken together, this suggests that the *Cga* TSS nucleosome might be evicted by Chd1 to create a depleted region at this promoter.

### H2A.Z modulates *Lhb* TSS and *Cga* +1 nucleosomes

Nucleosomes reconstituted on *Cga* and *Lhb* showed H3/H4 breaking forces in the order of 30 pN, suggesting that they constitute an important obstacle that must be modulated to allow access of the transcription machinery to the DNA. Notably, in virtually all eukaryotes, the +1 positioned nucleosome presents the highest barrier to transcription, as compared with the other nucleosomes further downstream^36^. This nucleosome is highly enriched by histone variant H2A.Z, which was recently proposed to be involved in lowering the barrier to Pol2 elongation and decreasing Pol2 stalling in *Drosophila*^36^.

**Figure 4:**
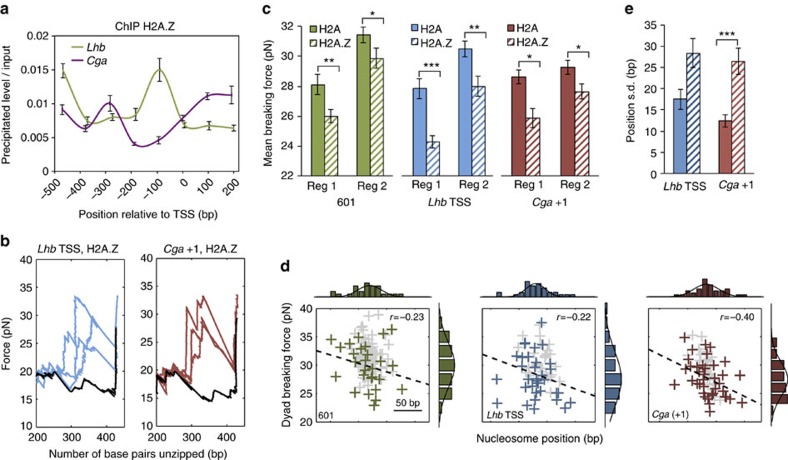
H2A.Z modulates *Lhb* TSS and *Cga* +1 nucleosomes. (**a**) ChIP for H2A.Z was carried out in LβT2 cells, using the same sets of primers as in Fig. 1a,b. Data are analysed and presented as in Fig. 1d; *n*=4. (**b**) Typical unzipping curves for *Lhb* TSS (blue) and *Cga* +1 (red) nucleosomes reconstituted with H2A.Z, each with its corresponding naked DNA sequence (black). (**c**) Breaking force for region 1 and region 2 interactions for 601 (dashed green), *Lhb* TSS (dashed blue) and *Cga* +1 (dashed red) nucleosomes, reconstituted with H2A.Z. For comparison, the H2A nucleosome data from Fig. 2 are also shown (solid colours). Data shown as mean±s.e.m., *n*=27, 34 and 38; *P*=0.002, 0.02, 0.001, 0.002, 0.004 and 0.04, two-sample Kolmogorov–Smirnov test. *P<0.05, **P<0.01, ***P<0.001. (**d**) Two-dimensional scatter plots of region 2 breaking force versus position, for all the individual measured traces for H2A.Z reconstituted 601 (left, green), *Lhb* TSS (middle, blue) and *Cga* +1 (right, red) nucleosomes. The data for the same sequences reconstituted with H2A (Fig. 2f) are also shown in the background (grey). (**e**) Positional dispersion of the *Lhb* TSS and *Cga* +1 nucleosomes reconstituted with H2A.Z (dashed blue and dashed red, respectively), and for comparison nucleosomes on the same sequences but reconstituted with H2A. Data shown as region 2 position s.d.±s.e., *n*=34, 38; *P*=9 × 10^−5^, two-sample Ansari–Bradley test. ***P<0.001.

To test whether H2A.Z is present in the +1 nucleosomes of *Cga* and *Lhb* we performed ChIP in gonadotrope cells, with an antibody against H2A.Z, followed by qPCR. These experiments revealed that the +1 nucleosome on *Cga* is marked by H2A.Z, as is the nucleosome that is positioned upstream of the NDR, centred around −300 bp. Interestingly, H2A.Z did not appear enriched on the *Lhb* +1 nucleosome, but instead localized immediately upstream of the TSS, at the position of the *Lhb* TSS nucleosome (Fig. 4a).

To understand the function of H2A.Z on *Cga* +1 and *Lhb* TSS DNA sequences, we reconstituted nucleosomes on these regions of DNA using octamers containing H2A.Z/H2B dimers (Supplementary Fig. 2), and subjected them to unzipping analysis using the optical tweezers. The unzipping signatures of H2A.Z-containing nucleosomes showed similar structural features as nucleosomes reconstituted with the canonical histones, with two prominent regions of strong histone–DNA interactions (Fig. 4b). However, for both DNA sequences tested, the mean breaking force of region 1, which corresponds to interactions of the H2A.Z/H2B dimer with the DNA, was reduced as compared with that seen for nucleosomes containing the canonical H2A (*P*=0.0008 and 0.004 for *Lhb* and *Cga*, respectively; two-sample Kolmogorov–Smirnov test; Fig. 4c). Strikingly, the mean breaking force of region 2, which corresponds to interaction of the H3/H4 tetramer with the DNA, was also reduced for both DNA sequences tested (*P*=0.002 and 0.04; two-sample Kolmogorov–Smirnov test; Fig. 4c,d). The nucleosomes reconstituted with H2A.Z/H2B-containing octamers on the 601 sequence showed similar reduction of breaking forces for regions 1 (*P*=0.001; two-sample Kolmogorov–Smirnov test) and 2 (*P*=0.02; two-sample Kolmogorov–Smirnov test). This suggests a universal mechanism of action for H2A.Z, in which it weakens the interactions between both the H2A.Z/H2B dimers and the H3/H4 tetramer with the DNA. Reconstitution with H2A.Z also resulted in a small shift of ∼10 bp upstream, in the mean position of the *Lhb* TSS nucleosome (*P*=0.02; two-sample Kolmogorov–Smirnov test), although there was no significant change in the mean position of nucleosomes on the *Cga* +1, or 601 sequence (Supplementary Fig. 7).

**Figure 5:**
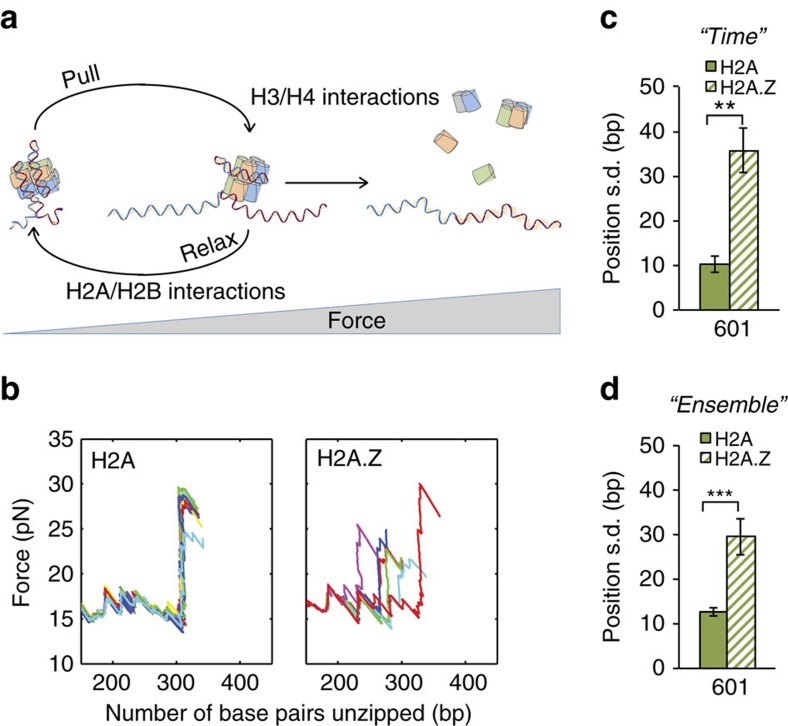
H2A.Z modulates the mobility of nucleosomes. (**a**) Experimental scheme for repetitive disruption of region 1. (**b**) Typical results for the repetitive disruption of the first interaction region in 601 nucleosomes containing H2A (left) and H2A.Z (right). (**c**) Positional dispersion for H2A- and H2A.Z-containing nucleosomes, reconstituted on 601 DNA, calculated as the standard dispersion over time, for the repetitive probing of an individual nucleosome. Data shown are the mean s.d.±s.e.m., *n*_nucleosomes_=5 and 4; *n*_repetitions_=5–36; *P*=0.007, Kolmogorov–Smirnov test. **P<0.01. (**d**) Positional dispersion calculated as the standard dispersion in the position of the ensemble of Fig. 4d±s.e., *n*=28 and 27; *P*=9 × 10^−5^, two-sample Ansari–Bradley test. ***P<0.001.

Our data also suggest an additional mechanism through which H2A.Z modulates the chromatin structure, as the dyad positional dispersion was significantly increased in all of the measured nucleosomes (Fig. 4d,e). This suggests that H2A.Z incorporation not only reduces nucleosome stability but also affects the histone octamer positioning on DNA. Interestingly, the source for the measured variability in position could be the result of two extreme scenarios, or a combination of them. One possibility is that the positional dispersion described above is the result of a ‘frozen' distribution of nucleosome positions in the ensemble created, for example, during reconstitution. Alternatively, it might be the result of nucleosomes dynamically repositioning on a timescale relevant to our experiments, in which case the position we measure is a ‘snapshot' of their dynamics. To clarify this question, we exploited the fact that breaking of the H2A/H2B interaction region is a reversible process, such that if the force is relaxed after the disruption, the interaction forms again. Hence, we subjected nucleosomes reconstituted on the 601 sequence to multiple cycles of unzipping of the H2A/H2B region followed by force relaxation to allow re-zipping, with a total cycle time of 30 s (Fig. 5a). These experiments showed that the position of single H2A.Z-containing nucleosomes exhibits a significantly larger dispersion (over time) as compared with the ones reconstituted with H2A (Fig. 5b,c). Remarkably, the dispersion ‘over time' as measured in these experiments (Fig. 5c) is in good agreement with the dispersion ‘over the ensemble' of the previous experiments (Fig. 5d), both for H2A and H2A.Z nucleosomes. Hence, we conclude that the measured dispersion in position is a result of the dynamics (that is, the mobility) of the nucleosomes, and that H2A.Z has a significant effect on this mobility. Finally, since H2A.Z has a strong effect on the dispersion even for the 601 sequence, selected for its strong positioning properties, it appears that although both the sequence of DNA and the presence of H2A.Z affect the mobility of nucleosomes, H2A.Z is a stronger determinant.

### H2A.Z modulates transcription through the *Cga* +1 nucleosome

**Figure 6:**
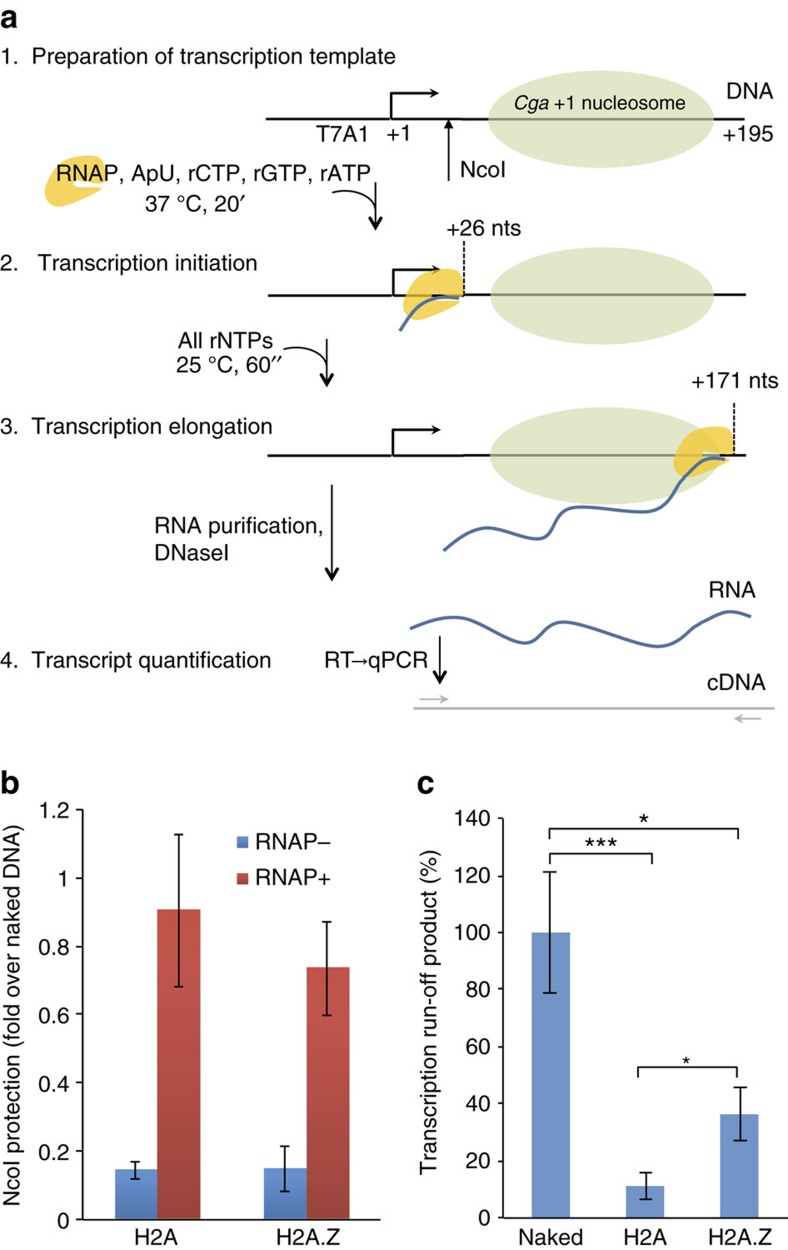
*In vitro* transcription through the *Cga* +1 nucleosome. (**a**) Description of experimental procedure for *in vitro* transcription experiments. (**b**) To rule out the possibility of different transcription initiation efficiency between the samples, we exploited the restriction enzyme site of NcoI, located at +25 on the transcription template, relative to the TSS. RNAP should protect DNA from NcoI digestion if it is stalled at +26. To quantify the degree of protection, DNA was purified and subjected to qPCR with specific primers spanning the transcription construct. (**c**) Naked and nucleosomal *Cga* +1 DNA were ligated to DNA containing a T7A1 promoter and subjected to *in vitro* transcription for 1 min. The RNA was purified, DNase I treated and reverse transcribed. RNA levels were quantified using qPCR with specific primers. Data shown as mean±s.e.m., *n*=9–10; *P*=0.01, 0.04 and 5 × 10^−4^, two-sample Student's *t*-test. *P<0.05, ***P<0.001. nts, nucleotides.

Since the +1 nucleosome in *Cga* is enriched with H2A.Z, we wondered whether the distinct properties conferred to this nucleosome by its presence might affect the efficiency of transcription. To that end, nucleosomes reconstituted on *Cga +*1 DNA and ligated to a T7A1 promoter were subjected to a single turnover, *in vitro* transcription assay using *E. coli* RNA polymerase (RNAP), which utilizes the same mechanism for transcription through a nucleosome *in vitro* as eukaryotic Pol II (ref. 37; Fig. 6a). Protection of a NcoI site located +25 relative to the TSS allowed us to quantify the relative efficiency of RNAP initiation (Fig. 6b), whereas the amount of run-off transcript detected by qPCR using specific primers allowed us to measure the percentage of RNAPs that were able to successively pass through the nucleosome (Fig. 6c). Nucleosomes reconstituted using canonical H2A allowed 11±5% run-off passage (mean±s.e.m.; Fig. 6c), consistent with previously published results^37^,^38^. Notably, although the initiation efficiency was not significantly affected (Fig. 6b) nucleosomes reconstituted with H2A.Z showed a significantly higher 36±10% run-off passage (*P*=0.04, two-sample Student's *t*-test; Fig. 6c), suggesting that the sensitivity of H2A.Z nucleosomes to mechanical force and their increased mobility, directly lead to an increased efficiency of transcription through these nucleosomes.

## Discussion

In our work, we aimed to elucidate which factors contribute to shaping the architecture of chromatin at the promoters of the *Cga* and *Lhb* genes, and the mechanism by which their expression levels are regulated. Both of these genes are expressed in gonadotropes but, since only *Lhb* is unique for LH, basal expression levels of *Cga* are much higher. Our results indicate that the structure of chromatin at the gene promoters plays an important role in establishing this large difference in expression. Thus, these two genes comprise a convenient model to study the alternative ways through which the cell regulates expression via the structure of promoter chromatin. The combination of *in vivo* mapping of the position and composition of promoter nucleosomes, with *in vitro* single-molecule biophysical characterization of their properties, allows us to formulate a model for the regulation of these genes.

**Figure 7:**
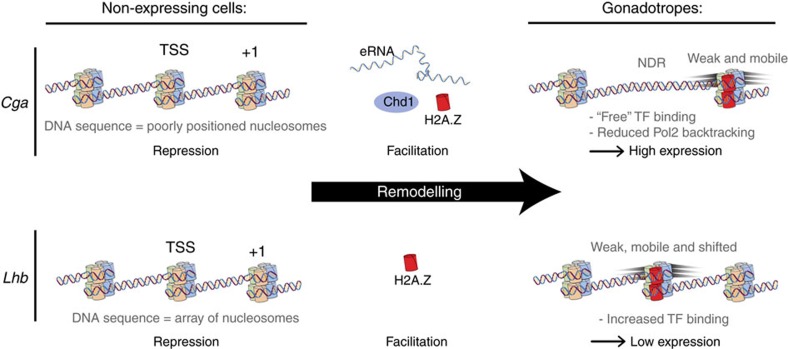
Proposed model for the differential regulation of *Lhb* and *Cga* through distinct modifications of their promoter chromatin. In non-expressing cells, the regulatory regions of both *Cga* and *Lhb*, which include binding sites for important TFs, are covered by nucleosomes, and the genes are repressed. In gonadotropes, these genes are remodelled in different ways, tailored to achieve a moderate expression of *Lhb* and a much higher expression of *Cga*.

Our results show that these two genes are regulated by distinct strategies, which exploit the sequence of DNA and the identity of the histone proteins (Fig. 7): in the case of the *Cga*, the nucleosome located at the TSS that blocks TF binding in non-expressing cells appears evicted by remodelling machinery in gonadotropes to create an NDR that completely exposes TF-binding sites. The +1 nucleosome is remodelled with H2A.Z to achieve a weaker and more mobile nucleosome. In the case of *Lhb*, for which a lower level of expression is required, a milder remodelling is observed. The TSS nucleosome is not evicted, but remodelled with the variant H2A.Z resulting in a weaker nucleosome. This nucleosome is slightly shifted from the position of a canonical H2A nucleosome on the same sequence, and is also more mobile.

The effect that the underlying sequence of DNA has on the properties of nucleosomes has been the subject of numerous studies. The nucleosome's stability is affected by the formation of specific DNA–histone interactions and by the sequence-dependent mechanical properties of DNA, in particular its curvature, flexibility and twist^39^. Previous works have shown that the sequence has an important effect on the positioning, structure and stability of nucleosomes^40^,^41^,^42^, and, recently, it was demonstrated that the sequence can also affect the dynamics of the nucleosome's local conformational transitions^18^. However, the importance of these effects in the context of real, transcribed genes has not been fully elucidated. In our work, we did not observe significant differences in the breaking force of nucleosomes reconstituted on the natural sequences probed, indicating that all of them can support the formation of stable nucleosomes, which can block TF-binding sites or perturb the ability of Pol2 to elongate. Significant differences were observed for the mobility of nucleosomes, with those at TSS sequences being more mobile than +1 nucleosomes. This increased mobility at the TSS nucleosomes could serve to maintain a basal level of DNA accessibility, allowing the recruitment of remodellers or pioneer TFs.

Notably, in all of the DNA sequences tested, incorporation of H2A.Z resulted in lower breaking forces and increased mobility. Although this histone variant is only ∼60% identical in sequence, the overall structure of the H2A.Z-containing nucleosome has a high degree of similarity to that of nucleosomes containing the canonical histone^43^. Significant differences are, however, in domain L1, important for the interactions between the two H2A/H2B dimers within the nucleosome, and the C-terminal docking domain, responsible for their interaction with the H3/H4 tetramer. The loss of hydrogen bonds between H2A.Z and H3/H4 is expected to weaken the interactions between H2A.Z/H2B and H3/H4, and therefore may be the source of the decreased stability (that is, decreased breaking force) of the H2A.Z nucleosomes. In addition, since residues at H2A's C terminus make stable hydrogen bonds with the DNA, it is possible that the increased mobility we observe here is the result of the absence of these bonds in H2A.Z-containing nucleosomes. Notably, truncation of H2A C-terminal domain has been reported to increase the thermal mobility of nucleosomes^44^.

Selective incorporation of H2A.Z into nucleosomes positioned at different DNA regions will affect various stages of mRNA transcription, thus establishing distinct expression patterns. The incorporation of H2A.Z into TSS nucleosomes is expected to influence greatly the initiation of transcription. The dynamic equilibrium model of Polach and Widom and their data^45^ indicate that the equilibrium constant for DNA accessibility is a function of the distance of a binding site from the nucleosome dyad, and that it varies by as much as four orders of magnitude from the dyad to the outer 70 bp. Hence, a dyad position shift as small as 10 bp can have a major effect (∼10-fold) on the binding equilibrium constant for a specific TF. The proximal ∼140 bp on the *Lhb* promoter where the TSS nucleosome is positioned are remarkably conserved among species, and harbour functional binding sites for tissue-specific TFs: two binding sites of steroidogenic factor-1 (Sf1) and one binding site of paired-like homeodomain 1 (Pitx1), both of which are required for gonadotrope differentiation and *Lhb* expression^46^. Modulation of the nucleosome on this sequence with H2A.Z, which induces a 10 bp position shift and also higher nucleosome mobility may increase the transient exposure of Sf1- or/and Pitx1-binding sites, allowing them to serve as ‘pioneer' TFs for further recruitment of chromatin remodellers^2^. More generally, the higher mobility induced by H2A.Z, which can increase the time-averaged exposure of some TF-binding sites, provides a milder control of TF binding as opposed to nucleosome eviction, and may allow moderate transcription initiation and relatively low expression levels, as is the case for *Lhb*. Interestingly, since higher mobility can also reduce the time-averaged exposure of other TF-binding sites, our results help shed light on the controversy between the repressive and activating roles of H2A.Z (ref. 47).

The incorporation of H2A.Z into the +1 nucleosome, as observed for *Cga*, would likely affect the early elongation phase. While every nucleosome represents a barrier for transcriptional elongation^48^,^49^ and induces Pol2 pausing, the +1 nucleosome creates an ∼3 times higher barrier than downstream nucleosomes^36^. Remarkably, stalled Pol2 at the nucleosomal boundary was detected in 50% of human genes^50^ and correlated with well-positioned +1 nucleosomes. When Pol2 encounters the nucleosomal barrier, ∼8–13 bp within the +1 nucleosome^36^, it backtracks allowing the octamer to regain full contact with the DNA^51^. In general, Pol2 recovers from the backtracked state by diffusing back into alignment of its active site with the 3′-end of the transcript. However, the newly formed contacts between the octamer and DNA prevent the recovery of Pol2 from its backtracked state. Since the diffusion process has no chemical energy input, Pol2 clearly lacks the ability to actively disrupt the nucleosome to reach alignment. Hence, the recovery is a passive process, a ‘Brownian ratchet' whereby Pol2 rectifies spontaneous wrapping/unwrapping dynamics of the nucleosome^19^,^52^. In the framework of this mechanism, the recovery might be expected to be affected not only by the wrapping dynamics of a nucleosome at a fixed position on the DNA but also by the repositioning of the nucleosome as a whole. Indeed, it has been shown that the nucleosomal barrier is relieved by ISW2, an ATP-dependent chromatin remodeller, which translocates the nucleosome over a short distance^49^. Moreover, *sin* mutations, which do not significantly alter the structure of nucleosomes but increase their mobility, have been shown to rescue defects in SWI/SNF action^53^, while deletion of H2A.Z in *Saccharomyces cerevisiae* strongly increased the need for SWI/SNF^54^. Notably, the dispersion measured here for H2A.Z nucleosomes is similar to the dispersion reported previously following the action of SWI/SNF^25^. Taken together, this suggests that the incorporation of H2A.Z nucleosomes serves to alleviate the strong +1 nucleosomal barrier via the increased nucleosome mobility reported here, which facilitates recovery from the backtracked state. Providing further support for this idea, our *in vitro* transcription assay shows that elongation through a *Cga* +1 DNA template, reconstituted with an H2A.Z-containing nucleosome, is ∼3 times more efficient than elongation on the same template with an H2A nucleosome (Fig. 6). Interestingly, the presence of H2A.Z in *Lhb* TSS and *Cga* +1 nucleosomes suggests that the gonadotrope cell takes advantage of the same physical property (that is, the increased nucleosomal mobility) to fulfil two distinct roles in regulating these genes.

How are DNA sequence and H2A.Z presence integrated into a regulation mechanism? Nucleosomal occupancy in the non-expressing MEF cells has a high degree of similarity with the predicted nucleosome-positioning profiles suggesting that in these cells the positioning is determined mainly by the sequence of DNA. In gonadotropes the occupancy map is very different from the prediction, indicating that the sequence is not the sole or main determinant of their positioning. As seen from the prediction and the mapping of MEFs, the sequence of *Lhb* imposes well-localized nucleosomes, that is, an ordered nucleosome array, so both the TSS and +1 nucleosome positions are dominated by the sequence. This structure blocks TF binding and imposes severe obstacles to transcription so, in gonadotropes, the TSS nucleosome is remodelled with H2A.Z to lower these obstacles. Hence, the efficiency of transcription is dictated by a competition between the repressive characteristics of the sequence and the facilitating properties of H2A.Z (Fig. 7). Also for *Cga* the sequence induces a structure that blocks TF-binding sites, but in this case it determines a poor localization and no nucleosomal array, and hence possibly a lower obstacle for transcription. In gonadotropes, this structure is modified in two ways: the TSS nucleosome is evicted resulting in an NDR; and the +1 nucleosome is remodelled with H2A.Z to achieve a weaker, more mobile nucleosome that facilitates transcription. This represents a complex mechanism for the regulation of the expression of *Cga* (Fig. 7): for the initiation of transcription, the repressive effect of the sequence (which stimulates the formation of a TSS nucleosome covering the regulatory regions) is overcome via Chd1-mediated nucleosome eviction. For the elongation phase, a competition between the repressive properties of the sequence and the facilitating effect of H2A.Z results in reduced backtracking and efficient transcription. A general feature of these regulation strategies is a competition between DNA sequences that promote the formation of stable nucleosomes, and have therefore a repressive action of various strengths, and remodelling of the nucleosomes, either by evicting them or introducing H2A.Z, thus achieving a facilitating action. It seems that such a strategy, where repression is achieved by a ‘passive' mechanism, that is, exploiting the binding affinity to DNA, and the activation in the specific cell types is achieved by active remodelling, is an efficient use of cellular resources.

The structure and dynamics of promoter chromatin, the most basic layer in the multilayer regulation of genes, are shaped in a gene-specific and cell-specific way through the effect of numerous factors. These include also the effect of long-range features of the chromatin, such as the effect of an eRNA transcribed distally as shown here and the specific recruitment of chromatin remodellers. However, all these effects converge eventually into creating a chromatin structure (that is, nucleosomes' positions, compositions and chemical modifications) whose properties then control the rate and fate of Pol2 transcription. Hence, to tailor the expression levels of different genes, while also optimizing the use of cellular resources, the cell can utilize combinations of factors and the properties dictated by them. Our results, although specific for the two genes studied, highlight how DNA sequence, the usage of an alternative histone variant and remodelling machinery act synergistically to control the expression of genes via the properties of their promoter chromatin.

## Methods

### Cell culture

Murine gonadotrope-derived αT3-1 and LβT2 cells (a kind gift from P. Mellon, UCSD), sieRNA αT3-1 cells^29^ and MEF cells (a kind gift from Arnon Henn, Technion) were cultured in minimum essential medium supplemented with 0.1 mM non-essential amino acids and 1 mM sodium pyruvate (αT3-1 and MEF cells), or Dulbecco's modified Eagle's medium containing 4.5 g l^−1^ glucose (LβT2 cells), to which 10% fetal calf serum, 10 mM HEPES, 100 U ml^−1^ penicillin and 100 μg ml^−1^ streptomycin were added (all Biological Industries, Bet Ha'Emek, Israel). The gonadotrope-derived αT3-1 and LβT2 cell lines have been authenticated in our lab to express the cell-specific gonadotropin genes: *Cga* abundantly in both cell lines; and *Lhb* at very much higher levels in the latter. This pattern of gene expression is constantly monitored in our lab by qPCR. All cell lines are regularly tested for mycoplasma contamination.

### MNase mapping of nucleosome positions

For MNase experiments, 5 × 10^6^–1 × 10^7^ cells were washed twice and then collected in cold PBS. After centrifugation (1,000*g* for 5 min), cells were permeabilized with 0.03% Triton X-100 and 10% fetal calf serum in PBS and incubated for 10 min at 37 °C. After centrifugation (1,000*g* for 5 min), cells were resuspended in reaction buffer (150 mM sucrose, 50 mM Tris·Cl (pH 7.5), 50 mM NaCl and 2 mM CaCl_2_). The chromatin was digested with MNase (2,000 gel units; New England Biolabs) for 15 min and quenched on ice with EGTA to a final concentration of 25 mM, resulting in predominantly ∼150 bp fragments corresponding to mono-nucleosomes (Supplementary Fig. 1c).

The samples were centrifuged at 20,000*g* for 10 min and purified using a PCR purification kit (28106; Qiagen). For low-coverage nucleosome position maps, MNase-digested DNA was subjected to real-time PCR with primers designed according to the predicted nucleosome positions, amplifying ∼100 bp non-overlapping fragments of DNA regions between −550 and +250 bp of *Cga* and *Lhb* genes (Supplementary Table 1). For high-coverage nucleosome position maps, primers generating amplicons of ∼70 bp with 10–20 bp overlap between neighbouring amplicons were used (Supplementary Table 2). The amount of DNA amplified after MNase digestion for each amplicon was plotted against genomic coordinates for the centres of each amplicon, and normalized to undigested sonicated genomic DNA, to create average nucleosome position maps for both *Cga* and *Lhb* genes. As a negative control for each gene, a reaction was performed designed to amplify a 250 bp fragment.

### Chromatin immunoprecipitation

For ChIP experiments, 5 × 10^6^–1 × 10^7^ cells were crosslinked with 1% formaldehyde for 10 min, after which glycine (125 mM final concentration) was added for 5 min to quench the crosslinking. The cells were washed twice and then were collected in cold PBS. After centrifugation (1,000*g* for 5 min), cells were lysed with 750 μl lysis buffer (50 mM HEPES·KOH (pH 7.5), 140 mM NaCl, 1 mM EDTA 1% Triton X-100, 0.1% sodium deoxycholate, 0.1% SDS and protease inhibitors). To obtain average DNA fragments of <200 bp, cells then were sonicated 60 times (15 s pulses, 33% amplitude, 10 s between each pulse) using a Sonics Vibra-Cell sonicator, while submerged in ice water. The cell debris was pelleted (10,000*g* for 5 min), and 12.5% of supernatant was removed to serve as the input. The remainder was immunoprecipitated by adding lysis buffer (750 μl), primary antibody (3 μg) and protein G magnetic beads (20 μl; 1004D Novex), with overnight incubation on a roller at 4 °C. After three washes, the samples were eluted (1% SDS and 100 mM NaHCO_3_). The crosslinking in the immunoprecipitated and input samples was reversed by incubation at 65 °C overnight with the addition of 2 μl RNase A (0.5 mg ml^−1^), before purification using a PCR purification kit (28106; Qiagen). Real-time qPCR with gene-specific primers (Supplementary Table 1) was then used to measure the levels of DNA, and the levels in the immunoprecipitated samples were normalized to those of the input. IgG was used as a negative control for all of the experiments. Antibodies used in the ChIP experiments included H3 (AB1791), H2A (AB18255), H2A.Z (AB4174) and IgG (AB6721), all from Abcam, Pol2 (N-20; Santa Cruz), and Chd1 (4351; Cell Signaling). All the antibodies were used at a 1:250 dilution.

### Luciferase assays

For luciferase assays, cells were grown in 96-well plates and transfected using PolyJet *In Vitro* DNA transfection reagent (Signagen) with reporter gene luciferase constructs (200 ng) of the mouse *Cga* (−507 to +46) or the mouse *Lhb* (−755 to +6) promoter sequences in the pGL2 plasmid, and a simian virus 40 (SV40)-Renilla luciferase reporter (2 ng). After 48 h, luciferase assays were carried out using the Dual-Luciferase reporter assay kit (Promega) according to the manufacturer's instructions, and levels of firefly luciferase activity normalized to those of the Renilla.

### Measurement of mRNA levels by real-time PCR

Total RNA was extracted using TRIzol (Ambion), treated with DNase and reverse transcribed with random hexamers (Applied Biosystems) for qPCR using gene-specific primers (Supplementary Table 3), Absolute Blue SYBR-Green ROX Mix (Thermo Fisher) and the Illumina Eco Real-Time PCR as reported^29^. Amplicon levels were quantitated relative to standard curves comprising cDNA or genomic DNA and were normalized to RPL0P mRNA.

### DNA constructs for nucleosome reconstitution

DNA sequences used for *Cga* and *Lhb* nucleosome reconstitution were amplified by PCR from mouse genomic DNA. Sequences for 601 nucleosomes^33^ were amplified from a plasmid that was a generous gift from Daniela Rhodes (MRC, Cambridge, UK). Primers used for the amplification reactions are listed in Supplementary Table 4. The constructs were digested using DraIII-HF (New England Biolabs) overnight according to the manufacturer's instructions. A 10 bp hairpin (Sigma) was ligated to the construct using T4 DNA ligase (New England Biolabs), in a reaction with 1:10 molar excess of the hairpin, at 16 °C. The construct was subsequently digested overnight with BglI (New England Biolabs).

### Histone purification

Histones were purified under acidic conditions as reported for mammalian cells^55^, but with some modifications for bacterially expressed histones. Specifically, histones were co-expressed using pCOLADUET-1 (Novagen) for H3 and H4, and PETDUET-1 (Novagen) for H2A and H2B in *E. coli* BL-21-codonplus (DE3), grown for 5 h at 37 °C in LB and induced with 0.8 mM isopropyl-β-D-thiogalactoside at 18 °C overnight. A volume of 1 l of cells for each histone pair was collected (5,000*g* for 10 min) and washed twice with cold TBS+phenylmethyl sulfonyl fluoride. The pellet was snap-frozen in liquid nitrogen, thawed, resuspended with 15 ml of tris-buffered-saline (TBS)+phenylmethyl sulfonyl fluoride and 1 mg ml^−1^ lysozyme, (Sigma) and sonicated 10 times (15 s pulses, 33% amplitude, 10 s between each pulse) using a Sonics Vibra-Cell sonicator, while submerged in ice water. The samples were cleared at 20,000*g* for 10 min and the supernatant was collected and treated with 0.1 M H_2_SO_4_ for 1 h on ice. An equal volume of Tris·Cl (pH 8) was added, the samples were cleared again at 20,000*g* for 10 min and the supernatant was collected. Subsequently, 0.2 M NaCl, 2 mM EDTA and 1 mM dithiothreitol (DTT) were added to final volume of ∼30 ml and the sample was loaded onto a SulfoPropyl cation exchange column (Sigma). The histones eluted (in 2 M NaCl, 2 mM EDTA and 50 mM Tris·Cl (pH 8)) at the first two fractions and were concentrated using Amicon Ultra (Merck).

Histone dimers were mixed at a 2(H2A/H2B)_2_:1(H3/H4)_4_ molar ratio, 5 mM DTT was added and histones were snap-frozen with liquid nitrogen and kept at −80 °C. Histone purity and stoichiometry were verified using 12.5% SDS–PAGE stained with coomassie blue (Supplementary Fig. 2a).

### Nucleosome reconstitution by salt dialysis

Nucleosomes were reconstituted under conditions reported^56^. The DNA constructs (2.5 pmol) were mixed with increasing amounts of histones in 2 M NaCl, 10 mM HEPES·KOH (pH 7.4) and 1 mM EDTA. The 80 μl reaction was loaded into a Slide-A-Lyzer MINI dialysis unit (Thermo Scientific) and dialysed with constant stirring against 1 l of reaction buffer at 4 °C. All reaction buffers contained 10 mM HEPES·KOH (pH 7.4), 1 mM EDTA, 0.02% NaNH_3_ and various amounts of NaCl as follows: 2 M NaCl for1 h; 1.5 M NaCl for 1.5 h; 1 M NaCl for 3 h; 0.75 M NaCl for 2 h; and 0 M NaCl—overnight.

The reactions were analysed using 1% agarose gels in 0.2 × TBE, post stained with ethidium bromide (EtBr) (Supplementary Fig. 2b), and fractions with the highest purity were selected for single-molecule experiments. The integrity of nucleosomes was confirmed by the following observations: (1) dynamic light scattering (DLS) measurements revealed a hydrodynamic diameter in agreement with previous results^57^ (Supplementary Fig. 3a); (2) two interaction regions were observed in the unzipping curves, both in the forward (Figs 2 and 4, and Supplementary Fig. 4) and reverse (Supplementary Fig. 5) direction, as previously reported^8^,^25^,^34^; and (3) unfolding of the inner and outer wraps was observed in single-molecule stretching experiments, at forces in agreement with previous reports^20^,^21^,^23^,^58^ (Supplementary Fig. 3f).

At least three different nucleosome reconstitutions were performed for each nucleosome type. No significant difference was observed between the reconstitutions.

### DLS measurements

DLS measurements of the reconstituted nucleosomes (0.3 mg DNA per ml) were performed at room temperature with a Vasco DLS system (Cordouan Technologies), in reaction buffer (10 mM HEPES·KOH (pH 7.4), 1 mM EDTA and 0.02% NaNH_3_). Analysis of the data was performed using the nanoQ software (Cordouan Technologies).

### Molecular construct for single-molecule experiments

We generated two 600 bp DNA handles each incorporating a specific tag (digoxygenin and biotin) using commercially purchased 5′ modified primers in a standard PCR reaction (Supplementary Table 5). The other two primers were designed to contain repeats of three DNA sequences recognized by single-strand nicking enzymes: Nt.BbvCI and Nb.BbvCI (both from New England Biolabs) on the biotin-tagged handle and on the digoxygenin-tagged handle, respectively. The nicking enzymes generated 29-nucleotide complementary overhangs on each handle. Handles were mixed at equal molar ratios for DNA annealing, creating a ∼1,200 bp fragment of annealed DNA handles. A ∼250 bp dsDNA alignment segment with the sequence of the 601 DNA was prepared using commercially purchased primers in a standard PCR reaction (Supplementary Table 5), ligated to the handles and gel-purified (QIAquick 28706, Qiagen).

Reconstituted nucleosomes were ligated to DNA handles using a rapid ligase system (Promega) in 3:1 molar ratio, 30 min at room temperature. The full construct (that is, handles+alignement segment+nucleosome) was incubated for 15 min on ice with 0.8 μm polystyrene beads (Spherotech), coated with anti-digoxigenin. The binding reaction efficiency was verified in a pull-down assay using 1% agarose gel in 0.2 × TBE post stained with EtBr (Supplementary Fig. 2c) The reaction was then diluted 1,000-fold in unzipping buffer (10 mM Tris·Cl (pH 7.4), 1 mM EDTA, 150 mM NaCl, 1.5 mM MgCl_2_, 1 mM DTT, 3% v/v glycerol and 0.01% BSA). Tether formation was performed *in situ* (inside the experimental chamber) by trapping an anti-digoxigenin bead (bound by nucleosomes) in one trap, trapping a 0.9 μm streptavidin-coated polystyrene beads in the second trap and bringing the two beads into close proximity to allow binding of the biotin tag in the nucleosomal DNA to the streptavidin in the bead.

### Optical tweezers

Experiments were performed in a custom-made dual-trap optical tweezers apparatus, similar to the set-up used by Moffitt *et al*.^59^, but with a number of differences. Briefly, the beam from a 852 nm laser (TA PRO, Toptica) was coupled into a polarization-maintaining single-mode optical fibre. The collimated beam out of the fibre, with a waist of *w*_0_=4 mm, was split by a polarizing beamsplitter into two orthogonal polarizations, each directed into a mirror and combined again with a second polarizing beamsplitter. One of the mirrors is mounted on a nanometre-scale mirror mount (Nano-MTA, Mad City Labs). A X2 telescope expands the beam, and also images the plane of the mirrors into the back focal plane of the focusing microscope objective (Nikon, Plan Apo VC 60X, NA/1.2), ensuring that steering will not result in shifting from the objective aperture. Two optical traps are formed at the objective's focal plane, each by a different polarization and with a typical stiffness of 0.3–0.5 pN nm^−1^. The light is collected by a second, identical objective, the two polarizations separated by a polarizing beamsplitter and imaged onto two Position Sensitive Detectors (First Sensor). The position of the beads relative to the centre of the trap is determined by back focal plane interferometry^60^. Calibration of the set-up was done by analysis the fluctuation spectrum of the trapped beads^61^, which were sampled at 100 kHz.

Stretching of naked DNA (that is, no nucleosome) tethers was used to find the polymer-model parameters under our experimental conditions. Stretching to 15 pN was used to fit an extensible worm-like-chain model (XWLC) for the stretching of the dsDNA handles, and the data at forces above the unzipping of DNA were used to fit a worm-like-chain model for the released ssDNA.

### Data analysis

Data were digitized at a sampling rate fs=2,500 Hz, and saved to a disk. All further processing of the data was done with Matlab (Mathworks). From the measured and filtered tether extension and force, the stretching of the dsDNA handles at each time point was subtracted from the measured extension. Then, the extension was divided by the extension of two ssDNA bases (calculated from the measured force using the worm-like-chain model) to result in the number of unzipped base pairs.

To improve the accuracy of the experiments, a 250 bp naked DNA segment was ligated to the reconstituted chromatin samples, and unzipped before the nucleosome^16^. This segment was used to perform a correlation-based alignment of all traces in a group (that is, DNA sequence) to a single curve used as a master curve, allowing shifting of the traces (that is, redefining the position of zero extension) and stretching of up to 2%. In each trace, we detected the nucleosome by searching for forces 3 pN above the unzipping force of the naked DNA at the same position, and identified the interaction regions in windows defined relative to this position. Traces identified by their unzipping signature as naked DNA, as containing multiple tethers or those unusually noisy were excluded from the analysis.

Differences in breaking forces and nucleosome positioning were checked using a two-sample Kolmogorov–Smirnov test, a non-parametric test that does not require assuming normality of the data. Differences in the variance of the position were checked using the non-parametric two-sample Ansari–Bradley test. Differences were considered statistically significant if the calculated *P* value was no larger than 0.05.

### *In vitro* transcription

Transcription experiments were performed under conditions reported^37^. Briefly, 4 nM of transcription template (DNA containing a T7A1 promoter with the sequence 5′-TATCAAAAAGAGTATTGACTTAAAGTCTAACCTATAGGATACTTACAGCC-3′, ligated to *Cga +*1 reconstituted nucleosomes), was incubated with 0.2 U of *E. Coli* RNAP (New England Biolabs). Transcription was initiated and stalled by adding 250 μM ApU (Ribomed) and 50 μM rATP, rGTP and rCTP in transcription buffer (25 mM Tris-Cl, pH 8.0, 150 mM KCl, 4 mM MgCl_2_, 1 mM DTT, 3% (v/v) glycerol and 0.02% (v/v) BSA) for 20 min at 37 °C. To prevent multiple rounds of transcription, the reaction was diluted 10-fold and competitor DNA (50 bp DNA containing the T7A1 promoter) was added to 45 nM. Transcription was resumed at room temperature by addition of 1 mM of all four rNTPs for 1 min. The reactions were quenched by addition of 10 mM EDTA. RNA was purified using RNA clean and concentrator-5 kit (Zymo research) with subsequent on-column DNase I (Zymo research) treatment according to the manufacturer's instructions. After purification, RNase inhibitor (New England Biolabs) was added and samples were treated again with 1 U of DNase I (Invitrogen) to eliminate DNA template residuals. The RNA was then reverse transcribed using qScript (Quanta) and quantified using qPCR with primers specific for the full-length RNA product, Perfecta Sybr-Green ROX (Quanta) and the TProfessional Basic Thermocycler Real-time PCR (Biometra). To rule out the possibility of different transcription initiation efficiency between the samples, we exploited the restriction enzyme site of NcoI, located at +25 on the transcription template, relative to the TSS. RNAP should protect DNA from NcoI digestion if it is stalled at +26. To quantify the degree of protection, DNA was purified and subjected to qPCR with specific primers spanning the transcription construct.

### Data availability

The data that support these findings are contained within the article or Supplementary Information files, or available from the authors on request.

## Additional information

**How to cite this article:** Rudnizky, S. *et al*. H2A.Z controls the stability and mobility of nucleosomes to regulate expression of the LH genes. *Nat. Commun.* **7,** 12958 doi: 10.1038/ncomms12958 (2016).

## Supplementary Material

Supplementary Information Supplementary Figures 1-7 and Supplementary Tables 1-7
