# Supplementary material for: H2A.Z controls the stability and mobility of nucleosomes to regulate expression of the LH genes
Source: Nat Commun. 2016 Sep 22;7:12958. doi: 10.1038/ncomms12958 (PMC5036153; doi:10.1038/ncomms12958)
Supplement: Supplementary Information — Supplementary Figures 1-7 and Supplementary Tables 1-7 [file ncomms12958-s1.pdf]

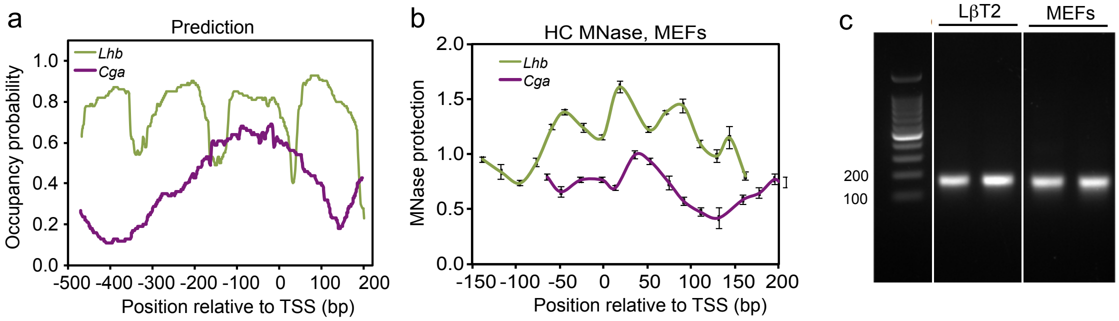

Supplementary Figure 1: Additional characterization of nucleosome positioning. (a) Sequence-based prediction of nucleosome positions for the promoters of *Cga* and *Lhb*. (b) High-coverage nucleosome maps were carried out in MEF cells, as in Fig 1c, and data is presented and analyzed similarly, n = 3–4. (c) Typical MNase digestion of genomic DNA of different cells used in the MNase experiments under the same conditions. Digested genomic DNA of LβT2 and MEFs was purified and run on a 2% agarose gel.

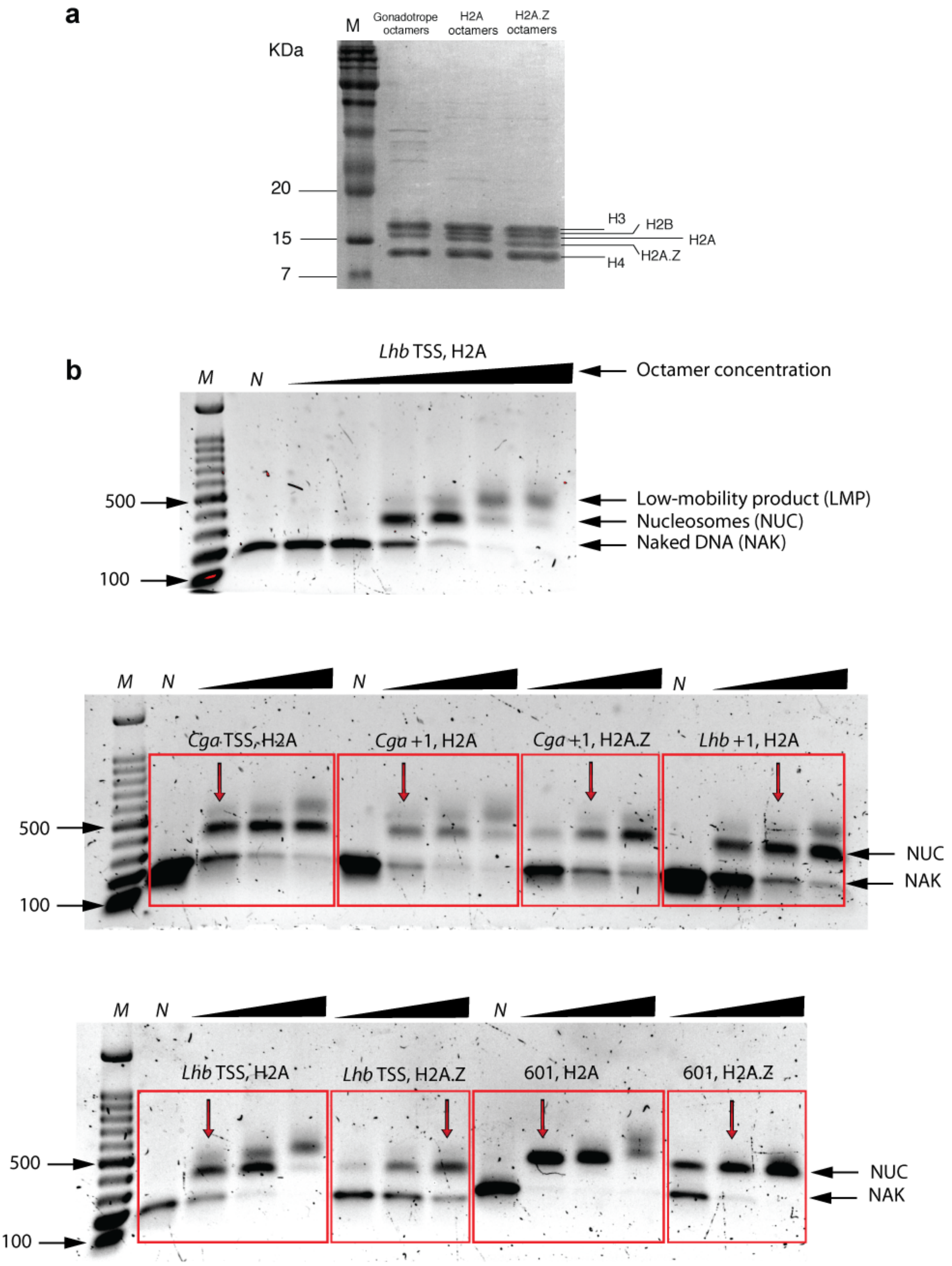

a
M
Gonadotrope octamers
H2A octamers
H2A.Z octamers
KDa
20
15
7
H3
H2B
H2A
H2A.Z
H4
b
Lhb TSS, H2A
M
N
Octamer concentration
500
100
Low-mobility product (LMP)
Nucleosomes (NUC)
Naked DNA (NAK)
M
N
N
N
Cga TSS, H2A
Cga +1, H2A
Cga +1, H2A.Z
Lhb +1, H2A
500
100
NUC
NAK
M
N
N
Lhb TSS, H2A
Lhb TSS, H2A.Z
601, H2A
601, H2A.Z
500
100
NUC
NAK

Supplementary Figure 2: Integrity of histones, nucleosomes and single-molecule optical-tweezers constructs. (a) Recombinant histones were expressed in bacteria separately as $(H2A/H2B)_2$ or $(H2A.Z/H2B)_2$ dimers and $(H3/H4)_4$ tetramers, mixed in a 2:1 (dimer:tetramer) molar ratio for reconstitution by salt gradient dialysis and run on 12.5% SDS-PAGE. Note that the H2A.Z band can be readily discriminated from that of H2A. Octamers purified from gonadotrope cells were used as a positive control. (b) Typical nucleosome reconstitution used for optical-tweezers experiments. DNA (221 bp for 601; 160 bp for *Lhb* and *Cga* constructs) was titrated with increasing amounts of histones for each reconstitution reaction. All the reconstitutions were performed at the same conditions and 1/8 of each reconstitution reaction was run on a 1% agarose gel post-stained with EtBr. "N" stands for naked DNA – with no histones. Three bands are seen in the gels, with intensities that vary depending on the concentration of histones used for the reconstitution: Naked DNA (NAK), nucleosomes (NUC), and a lower mobility product (LMP) that appears at high histone concentrations. We attribute this band to non-specific binding of histones to DNA, as indicated by their mobility and by their inability to efficiently ligate to the dsDNA handles, which suggests that the restrictions sites are covered by histones. To improve the purity of our nucleosomes, we chose samples (indicated by the red arrows) at an octamer concentration below the one that resulted in the nucleosome's band peak intensity. In this way, while we had a significant fraction of naked DNA (which can be easily identified in the optical tweezers), we had practically no traces with non-specific histone binding to DNA.

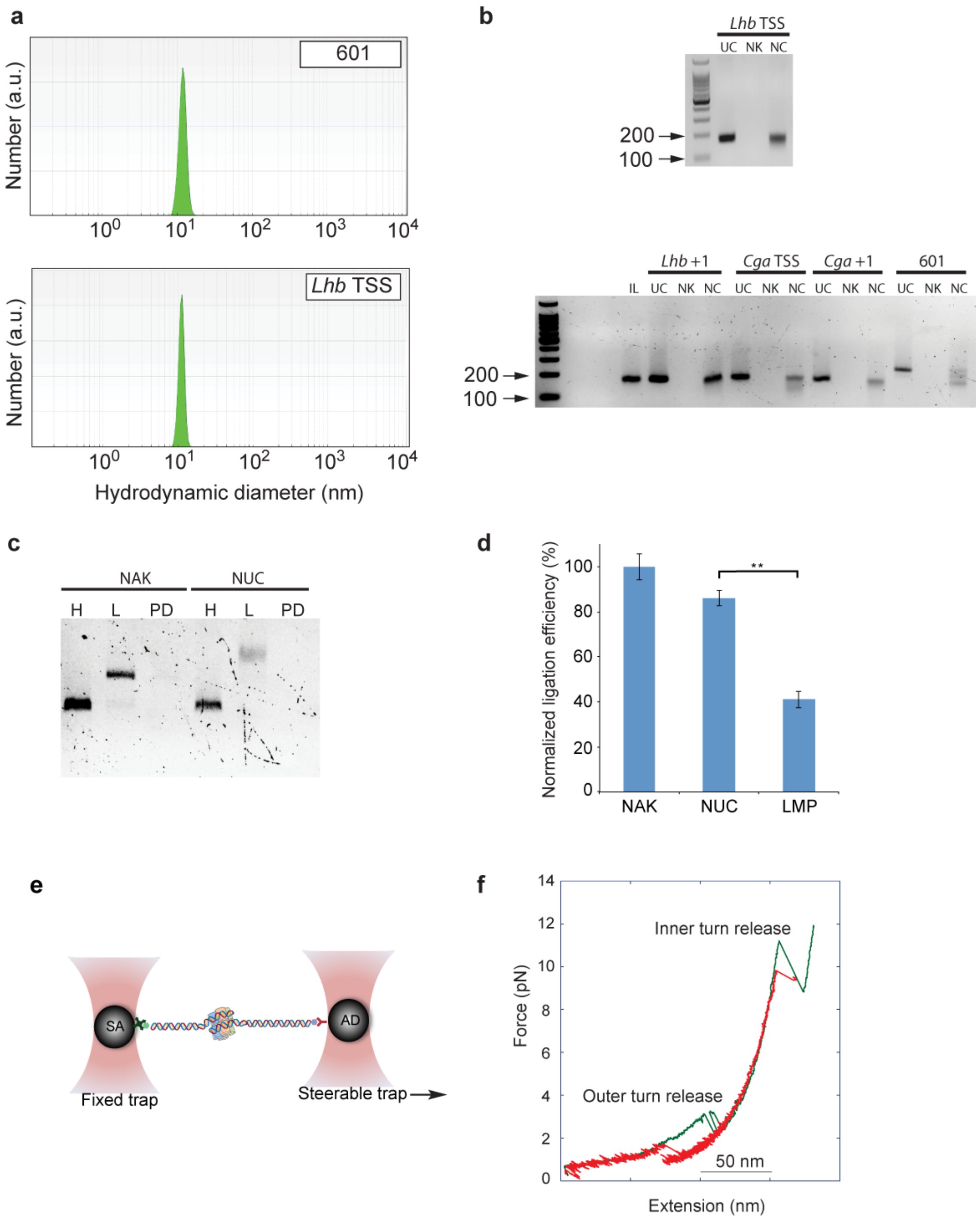

a
601
Number (a.u.)
10^0 10^1 10^2 10^3 10^4
Lhb TSS
Number (a.u.)
10^0 10^1 10^2 10^3 10^4
Hydrodynamic diameter (nm)
b
Lhb TSS
UC NK NC
200
100
Lhb +1
Cga TSS
Cga +1
601
IL UC NK NC UC NK NC UC NK NC UC NK NC
200
100
c
NAK
NUC
H L PD H L PD
d
Normalized ligation efficiency (%)
0 20 40 60 80 100
**
NAK NUC LMP
e
SA
AD
Fixed trap
Steerable trap
f
Force (pN)
0 2 4 6 8 10 12 14
Inner turn release
Outer turn release
50 nm
Extension (nm)

Supplementary Figure 3: Additional characterization of reconstituted nucleosomes. (a) Nucleosomes reconstituted on 601 DNA (upper panel) and *Lhb* TSS (lower panel) were subjected to Dynamic Light Scattering analysis. Measured hydrodynamic diameters were 10.7 ± 1.2 nm and 10.3 ± 1.4 nm (mean ± 90% C.I.), for 601 DNA and *Lhb* TSS, respectively. (b) A total of ~2.5 pmol of each reconstituted nucleosome was subjected to 20 gel units of MNase for 10 min at 37°C in reaction buffer. The DNA was purified using a PCR purification kit. The reaction products were run on a 2.5% agarose gel. UC = non-digested naked DNA construct, NK = digested naked DNA, NC = digested nucleosomes. (c) Reconstituted nucleosomes were ligated to dsDNA handles with a terminal digoxigenin, incubated with beads coated with antibodies against digoxigenin, pulled-down, and the supernatant was run on 1% agarose post stained with EtBr. H = dsDNA handles; L = ligation of handles and construct; PD = pull-down. (d) Ligation products were used as templates for a qPCR reaction, using a forward primer located in the dsDNA handle, and a reverse primer located in the reconstitution template. The results were normalized to the mean of the results obtained with naked DNA. NAK = naked DNA; NUC = nucleosome sample; LMP = sample at high octamer loading, with high concentration of LMP. The efficiency of ligation is 2.1-fold higher for NUC-rich samples as compared to LMP-rich samples ($p=9x10^{-4}$, two-sample Student's t-test.). (e) Reconstituted nucleosomes were ligated at both ends to ~600 bp dsDNA handles that allow to apply a stretching force on the construct. (f) Force-extension curves for 601 (green) and *Lhb* TSS (green) nucleosomes reveal the typical low-force opening of the outer turn, and high-force opening of the inner turn.

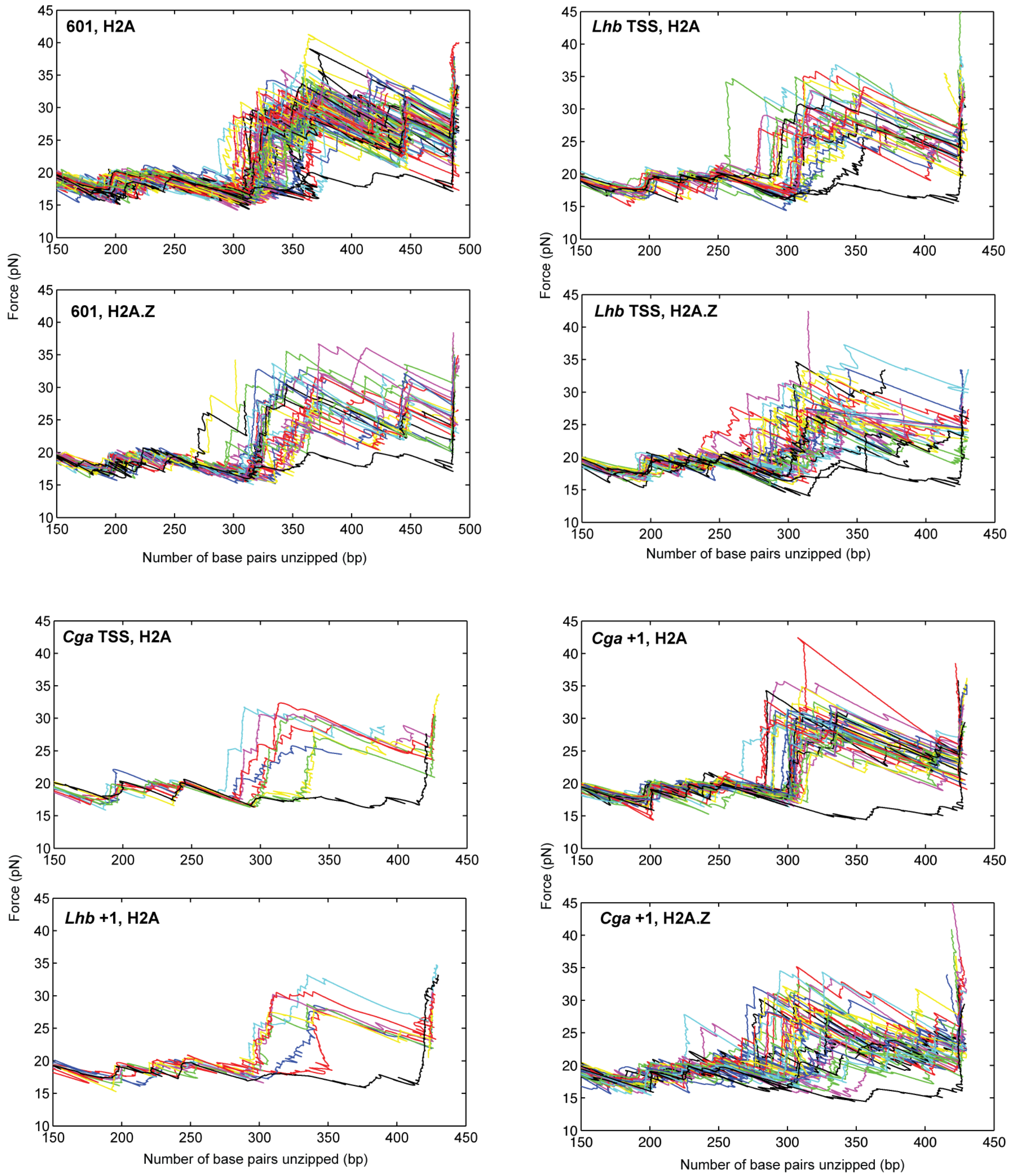

Supplementary Figure 4: Single-molecule unzipping curves. Unzipping traces for all of the nucleosomes measured (colored lines) together with a typical unzipping trace of their respective naked DNA (black). n= 88, 27, 28, 34, 7, 8, 37, 38, 27, 28. Total=322.

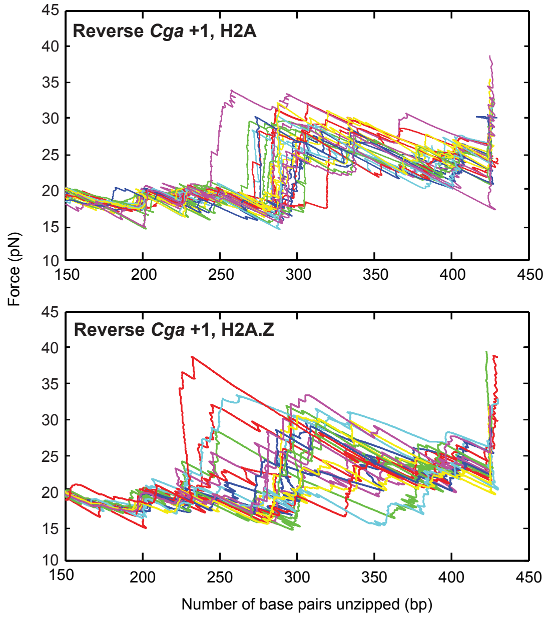

Supplementary Figure 5: Reverse unzipping curves. Representative unzipping traces for *Cga* +1 nucleosomes, ligated to the DNA handles in the reverse orientation. n=27, 28.

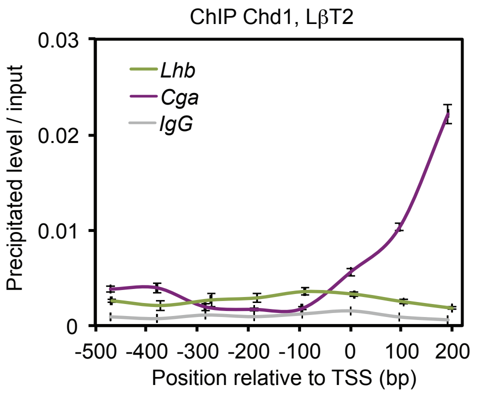

Supplementary Figure 6: **Chd1 is not located on the *Lhb* promoter.** ChIP for Chd1 was carried out in LβT2 cells expressing both *Cga* and *Lhb*. ChIP data is analyzed and presented as in Fig. 1d. Data are shown as mean ± SEM, n = 3.

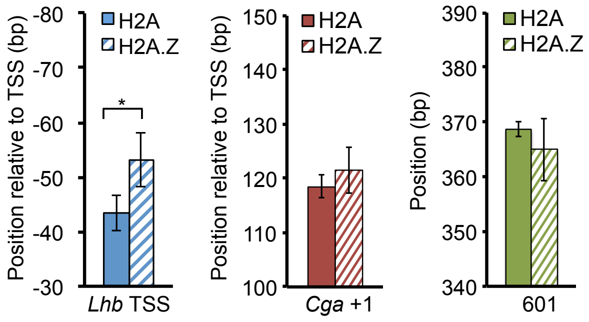

Supplementary Figure 7: H2A.Z effect on the nucleosome's position. Region 2 position for nucleosomes reconstituted with H2A (solid bars) and H2A.Z (dashed bars). Data shown as mean ± SEM, n≥34; For *Lhb* TSS, p=0.02; Kolmogorov-Smirnov test. *p<0.05.

Supplementary Table 1: Primers used for low-coverage MNase- and ChIP-qPCR

Number of amplicon	Amplicon center (relative to TSS)	Primer	Primer sequence (5'→3')
1	-470	*Cga* -525F	CACACCTGGACATATCTACTGT
		Cga -425R	AATTGCTCCAATTTCTTAAATTAAC
2	-379	*Cga* -429F	GGAGCAATTGTTTTATTTTTCTGT
		Cga -329R	ATTAGCTAAGTACCTGATATTTTCA
3	-284	*Cga* -334F	GCTAATTAAATGTGCTACTCTTAG
		Cga -235R	GGCCATTTTCTATGTAATATTATTG
4	-188	*Cga* -238F	GGCCAAATGCTCTCTCTTCA
		Cga -139R	TTGATCATATCACATTGCAACCC
5	-94	*Cga* -144F	GATCAATTGATGTCATGGTAATT
		Cga -45R	AATTGCATGTTCCCAGCTG
6	+1	*Cga* -50F	GCAATTTGTATTACTCTGCTGGT
		Cga +50R	GATTGT*CGA*TCTGAGGGGAA
7	+97	*Cga* +47F	GTGGTCACAAATATTTTACTCTTT
		Cga +147R	ATCACCTGCCCAGAACAC
8	+192	*Cga* +142F	CCACAGGAAACAATTTTCTGCATA
		Cga +242R	ACAGACCTCCCTCCAACTCA
1	-468	*Lhb* -518F	GACACACCCCTTACTTCCAGAG
		Lhb -419R	GGATGCCTGGATTGGGTCCAA
2	-372	*Lhb* -422F	ATCCTGATTAGGGGCTGGGAG
		Lhb -323R	GGCACAGCTTCAACCTTAGGTT
3	-273	*Lhb* -323F	CCTCCTATTTAGTTGTACCCAACCA
		Lhb -224R	TAAAGGGGAGGCCACTGCAG
4	-183	*Lhb* -233F	CTCCCCTTTACCTTGTTTCCCGT
		Lhb -134R	CAGGGACTAGCTCCAGTGTC
5	-89	*Lhb* -139F	TCCCTGGCTTCCCTGACCTTG
		Lhb -40R	TGGGGGTGGCAAGGCCACTA
6	+8	*Lhb* -42F	CCACAACCCGCAGGTATAA
		Lhb +58R	GATTGGGAAGTACCCTGGG
7	+105	*Lhb* +55F	AATCCCACCAAACCCATAGATG
		Lhb +154R	GTTTATCTCCTGTTTGAAGTCCAC
8	+200	*Lhb* +150F	TAAACAGATTTCATGGCTGGTGATG
		Lhb +249R	TCTACCCCTGACCTGGTTTTTC

Supplementary Table 2: Primers used for high-coverage MNase-qPCR

Number of amplicon	Amplicon center (relative to TSS)	Primer	Primer sequence (5'→3')
1	-65	*Cga* -100F	TGGGTAGAATTTACCCCTGATCC
		Cga -31R	CAGCAGAGTAATACAAATTGCAT
2	-49	*Cga* -83F	TGATCCCTGAGCTTATGTGC
		Cga -14R	CCCACTGTCTTTTATACCAG
3	-26	*Cga* -61F	CTGGGAACATGCAATTTGTA
		Cga +8R	GTGCCTGCAGTTAATAAAGTCT
4	-2	*Cga* -39F	ACTCTGCTGGTATAAAAGACAG
		Cga +34R	GGGGAACAATGTCTCTGGATT
5	+14	*Cga* -20F	CAGTGGGAGACTTTATTAACTGC
		Cga +48R	GATTGT*CGA*TCTGAGGGGAAC
6	+36	*Cga* -1F	CTGCAGGCACTGAAAAATCCA
		Cga +72R	TTGAGGGATGTGTTCTGGGCA
7	+54	*Cga* +19F	AGAGACATTGTTCCCCTCAG
		Cga +88R	CCTACCTCTGGACTTTTTGAG
8	+75	*Cga* +39F	AT*CGA*CAATCACCTGCCCAG
		Cga +111R	CCCCCAAATGATTTCATCATATTACC
9	+92	*Cga* +56F	CAGAACACATCCCTCAAAAAG
		Cga +127R	CTCTTTAATTAAATCCCCCCAA
10	+111	*Cga* +76F	GTCCAGAGGTAGGTAATATGATG
		Cga +146R	TGTGGTCACAAATATTTTACTC
11	+132	*Cga* +97F	TGAAATCATTTGGGGGGATT
		Cga +166R	TTATGCAGAAAATTCTTTCCTGT
12	+159	*Cga* +124F	AGAGTAAAATATTTGTGACCACAG
		Cga +194R	GAATGCCTAATTATTTTCCTACC
13	+178	*Cga* +143F	CACAGGAAAGAATTTTCTGCA
		Cga +214R	GCACCATAAAGAAAATTAATGAATGCC
14	+195	*Cga* +160F	TGCATAAATGTTGGTAGGAAAATAATTAGGC
		Cga +230R	TCCAACTCAGCCATGAGCAC
15	+209	*Cga* +172F	GGTAGGAAAATAATTAGGCATTCATT
		Cga +245R	TTCTACAGACCTCCCTCCAA
16	+231	*Cga* +196F	TTAATTTTCTTTATGGTGCTCATGG
		Cga +265R	A*CGA*TTGCCTTAGCTTACAG

Supplementary Table 2: Primers used for high-coverage MNase-qPCR (continuation)

Number of amplicon	Amplicon center (relative to TSS)	Primer	Primer sequence (5'→3')
1	-138	*Lhb* -173F	TGAGGCCAATTCACTGGGACAC
		Lhb -103R	TTGGGGG*CGA*GACACAGACAA
2	-117	*Lhb* -152F	ACTGGAGCTAGTCCCTGGCTT
		Lhb -81R	GGTAACCTAGACACTAATCTCTT
3	-96	*Lhb* -130F	TCCCTGACCTTGTCTGTGTC
		Lhb -61R	AAGTAGTGGCTACAGGCTTGG
4	-77	*Lhb* -111F	CGCCCCCAAAGAGATTAGTGT
		Lhb -42R	TGGGGGTGGCAAGGCCACTA
5	-57	*Lhb* -94F	GTGTCTAGGTTACCCAAGCCT
		Lhb -23R	GCTTTATACCTGCGGGTTGT
6	-44	*Lhb* -79F	AAGCCTGTAGCCACTACTTAG
		Lhb -9R	ACCTTGGGCACCTGGCTTTA
7	-23	*Lhb* -58F	TGGCCTTGCCACCCCCACAA
		Lhb +12R	CTCCATTCTTGATACCTTCCC
8	-1	*Lhb* -36F	CGCAGGTATAAAGCCAGGTGC
		Lhb +34R	TGGGCCCTACCATCTTACCT
9	+19	*Lhb* -17F	CCCAAGGTAGGGAAGGTATCA
		Lhb +54R	ATTGGGAAGTACCCTGGGC
10	+51	*Lhb* +16F	GAGGCTCCAGGTAAGATGGT
		Lhb +86R	AAGGCTGTCCATCTATGGGT
11	+71	*Lhb* +36F	AGGGCCCAGGGTACTTCCCA
		Lhb +106R	TCCTCCAACCCCCAGGTCAC
12	+91	*Lhb* +56F	ATCCCACCAAACCCATAGAT
		Lhb +126R	AGGAGACCCCCTCCTCTC
13	+111	*Lhb* +75F	TGGACAGCCTTGTGACCT
		Lhb +146R	TGTTTGAAGTCCACCAGCT
14	+130	*Lhb* +92F	TGGGGGTTGGAGGAGGGA
		Lhb +167R	CCATGAAATCTGTTTATCTCCTG
15	+144	*Lhb* +109F	AGAGGAGGGGGTCTCCTA
		Lhb +179R	GACCCATCACCAGCCATGA
16	+162	*Lhb* +127F	GCTGGTGGACTTCAAACAG
		Lhb +197R	ACAGAAACTGTCCTTCAAGAC
17	+180	*Lhb* +145F	GGAGATAAACAGATTTCATGGC
		Lhb +215R	GCACCCACTCAGTATAATACA
18	+202	*Lhb* +167F	TGGTGATGGGTCTTGAAGGA
		Lhb +237R	TGGTTTTTCCATCCCAGGT
19	+221	*Lhb* +185F	GACAGTTTCTGTATTATACTGAGT
		Lhb +257R	CTCACTCTACCCCTGACC

Supplementary Table 3: Primers used for mRNA quantification using qPCR

Gene	Primer (position relative to TSS)	Primer sequence (5'→3')
Cga	+97F	ATGGATTACTACAGAAAATATGCAG
	+196R	CCTGAATAATAAAGTCTCCATCAGG
Lhb	+200F	TGCCGGCTGCTTTGCCTCCT
	+396R	CAGGCCATTGGTTGAGTCCT
RPL0P	+96F	G*CGA*CCTGGAAGTCCAACTA
	+296R	ATCTGCTTGGAGCCCACAT

Supplementary Table 4: Primers used for PCR amplification of DNA sequences used for *Lhb, Cga* and 601 nucleosomes reconstitution

Gene/ sequence	Nucleosome	Incorporated restriction site	Primer (position rel. to TSS)	Primer sequence (5'→3')
Lhb	TSS	BglI	-130F	ATGGCCTTGCCGGCCCTGACCTTGTCTGTGTCTC
		DraIII	+23R	ATCACTGCGTGGAGCCTCTCCATTCTTGAT
	+1	BglI	+29F	ATGGCCTTGCCGG*CGA*TGGTAGGGCCCAGGGTA
		DraIII	+17R	ATCACTGCGTGAGACCCATCACCAGCCATG
Cga	TSS	BglI	-159F	ATGGCCTTGCCGGCTGCAATGTGATATGATCAATTGATG
		DraIII	-9R	ATCACTGCGTGAGTCTCCCACTGTCTTTTATAC
	+1	BglI	+39F	ATGGCCTTGCCGGCAT*CGA*CAATCACCTGCCC
		DraIII	+189R	ATCACTGCGTGCTAATTATTTTCCTACCAACATTTATGC
601 positioning sequence	*601*	BglI	F	ATGGCCTTGCCGGC*CGA*TGGACCCTATACGCGGCC
		DraIII	R	ATCACTGCGTGGAATT*CGA*TATCCC*CGA*GAAGG

Supplementary Table 5: Primers used for PCR amplification of DNA sequences used for optical tweezers handles preparation

Name	Primer/modification	Primer sequence (5'→3')
Biotin handle	F (5'-biotin)	GCTTTAATGCGGTAGTTTATCA
	R (Nt.BbVCI)	GCAGCATTAGGAAGCAGCCCAGGCATTAGGAAGCAGCCCAG
Dig handle	F (5'-phosphate/Nb.BbvCI)	GCATTAGGAAGCAGCCCAGGCTTTATTGCGGTAGTTTATCA
	R (5'-digoxigenin)	GCATTAGGAAGCAGCCCAG
601 alignment	F (BglI)	ATGGCCTGCACGGC*CGA*TGGACCCTATACGCGGCC
	R (DraIII)	ATCACTGCGTGGAATT*CGA*TATCCC*CGA*GAAGG

Supplementary Table 6: DNA sequences used for *Lhb, Cga* and 601 nucleosomes reconstitution

Nucleosome	DNA sequence (5' → 3')
Cga TSS (-9/-159)	TGCAATGTGATATGATCAATTGATGTCATGGTAATTATACCAAGTGCCATCCAA<br>TCACTGGGTAGAATTTACCCCTGATCCCTGAGCTTATGTGCAGCTGGGAACATG<br>CAATTTGTATTACTCTGCTGGTATAAAAGACAGTGGGAGACT
Cga +1 (+39/+189)	AT*CGA*CAATCACCTGCCCAGAACACATCCCTCAAAAAGTCCAGAGGTAGGTAA<br>TATGATGAAATCATTTGGGGGGATTTAATTAAAGAGTAAAATATTTGTGACCA<br>CAGGAAAGAATTTTCTGCATAAATGTTGGTAGGAAAATAATTAG
Lhb TSS (-128/+23)	CCTGACCTTGTCTGTGTCTCGCCCCCAAAGAGATTAGTGTCTAGGTTACCCAAG<br>CCTGTAGCCACTACTTAGTGGCCTTGCCACCCCCACAACCCGCAGGTATAAAG<br>CCAGGTGCCCAAGGTAGGGAAGGTATCAAGAATGGAGAGGCTC
Lhb +1 (+29/+179)	GATGGTAGGGCCCAGGGTACTTCCCAATCCCACCAAACCCATAGATGGACAGC<br>CTTGTGACCTGGGGGTTGGAGGAGGGAGAGGAGGGGGTCTCCTAGCTGGTGGA<br>CTTCAAACAGGAGATAAACAGATTTCATGGCTGGTGATGGGTCT
*601 (221 bp)**	TGCGTGGAATT*CGA*TATCCC*CGA*GAAGGTCGCTGTTCAATACATGCACAG**GAT**<br>**GTATATATCTGACACGTGCCTGGAGACTAGGGAGTAATCCCCTTGGCGGT**<br>**TAAAACGCGGGGGACAGCGCGTACGTGCGTTTAAGCGGTGCTAGAGCTTG**<br>**CTACGACCAATTGAGCGGCCTCGGCACCGGGATTCT**CCAGGGCGGCCGCGT<br>ATAGGGTCCATCGGCCG

(*) The canonical 601 sequence, composed of 150 bp, is marked in bold.

Supplementary Table 7: Quantification of the purity of reconstituted nucleosomes used in the optical tweezers experiments

Construct		NAK	NUK	LMP	Purity	
					From Gel	After ligation*
Lhb TSS	H2A	41%	53%	5%	91%	>96%
Cga TSS	H2A	41%	52%	7%	88%	>94%
Cga +1	H2A	39%	53%	8%	87%	>93%
Cga +1	H2A.Z	46%	46%	8%	85%	>92%
Lhb +1	H2A	32%	60%	8%	88%	>94%
Lhb TSS	H2A	35%	54%	10%	84%	>92%
Lhb TSS	H2A.Z	31%	61%	8%	88%	>94%
601	H2A	1%	98%	1%	99%	100%
601	H2A.Z	8%	90%	2%	98%	>99%

* Based on at least 2:1 enrichment as indicated in Supplementary Fig. 3d
